# Bottom-up growth of homogeneous Moiré superlattices in bismuth oxychloride spiral nanosheets

**DOI:** 10.1038/s41467-019-12347-7

**Published:** 2019-10-02

**Authors:** Lulu Liu, Yuanhui Sun, Xiaoqiang Cui, Kun Qi, Xin He, Qiaoliang Bao, Weiliang Ma, Jiong Lu, Hanyan Fang, Peng Zhang, Lirong Zheng, Liping Yu, David J. Singh, Qihua Xiong, Lijun Zhang, Weitao Zheng

**Affiliations:** 1 State Key Laboratory of Automotive Simulation and Control, State Key Laboratory of Superhard Materials, Key Laboratory of Automobile Materials of MOE, School of Materials Science and Engineering, Jilin University, Changchun, 130012 China; 2 College of Electronic Science & Technology and Institute for Advanced Study, Shenzhen University, Shenzhen, 518060 PR China; 3 Key Laboratory of Optoelectronic Devices and Systems of Ministry of Education and Guangdong Province, College of Optoelectronic Engineering, Shenzhen University, Shenzhen, 518060 PR China; 4 Department of Materials Science and Engineering, ARC Centre of Excellence in Future Low-Energy Electronics Technologies (FLEET), Monash University, Clayton, Victoria 3800 Australia; 5 Department of Chemistry, National University of Singapore, 3 Science Drive 3, Singapore, 117543 Singapore; 6 Department of Chemistry, Dalhousie University, Halifax, Nova Scotia B3H 4R2 Canada; 7 Beijing Synchrotron Radiation Facility, Institute of High Energy Physics, Chinese Academy of Sciences, Beijing, 100190 China; 8 Department of Physics and Astronomy, University of Maine, Orono, ME 04469 USA; 9 Department of Physics and Astronomy, University of Missouri, Columbia, MO 65211-7010 USA; 10 Division of Physics and Applied Physics, School of Physical and Mathematical Sciences, Nanyang Technological University, Singapore, 637371 Singapore

**Keywords:** Catalyst synthesis, Photocatalysis, Photocatalysis

## Abstract

Moiré superlattices (MSLs) are modulated structures produced from homogeneous or heterogeneous 2D layers stacked with a twist angle and/or lattice mismatch. Expanding the range of available materials, methods for fabricating MSL, and realization of unique emergent properties are key challenges. Here we report a facile bottom-up synthesis of homogeneous MSL based on a wide-gap 2D semiconductor, BiOCl, using a one-pot solvothermal approach with robust reproducibility. Unlike previous MSLs usually prepared by directly stacking two monolayers, our BiOCl MSLs are realized in a scalable, direct way through chemical growth of spiral-type nanosheets driven by screw-dislocations. We find emergent properties including large band gap reduction (∼0.6 eV), two-fold increase in carrier lifetime, and strongly enhanced photocatalytic activity. First-principles calculations reveal that such unusual properties can be ascribed to the locally enhanced inter-layer coupling associated with the Moiré potential modulation. Our results demonstrate the promise of MSL materials for chemical and physical functions.

## Abstract (web summary)

Expanding the range of available materials, methods for fabricating Moiré superlattices, and realization of new emergent properties are key challenges. Here the authors report a facile bottom-up synthesis of homogeneous Moiré superlattices based on a wide-gap 2D semiconductor, bismuth oxychloride.

## Introduction

Stacking atomically thin two-dimensional (2D) layers into artificial van der Waals heterostructures provides a flexible avenue for engineering physical properties of the 2D materials^1,2^. Moving toward a more generic 2D semiconductor material, such van der Waals stacking approaches provide the venue that electron quantum metamaterials can be rationally fabricated, with a length scale comparable to the wavelength of electron, distinct from the optical metamaterials^3^. The Moiré superlattice (MSL) is a particularly interesting case, consisting of a modulated structure produced from two homogeneous layers stacked with a twist angle^4–10^ or two heterogeneous layers incorporating a lattice mismatch and/or a twist angle^11–32^. The main characteristic of the MSL is the appearance of unique global structure periodicity and symmetry distinct from the constituent single layers. The resulting modulated electrostatic potential and strain field combined with the ubiquitous interlayer coupling, may give rise to distinct emergent physical properties. As demonstrated in the most studied case of graphene-based MSLs, these include band gap opening^12,13^, Fermi velocity renormalization^4,5^, cloned Dirac Fermions^13–16^, formation of quasicrystal^11,17^, dynamic band-structure tuning under compression^18^, Hofstadter-butterfly spectra^16,20–23^, fractional quantum Hall effect^22,23,33^, and superconductivity^12,19^. The rich diversity of 2D materials^1,3^ as building blocks provides potential for realizing a great variety of unique properties and functionalities if more MSLs can be made from them. However, thus far homogeneous MSLs have been obtained experimentally in stacked graphenes^4–8^. Reports of the heterogeneous MSLs are limited to the bilayer systems of graphene and hexagonal boron nitride^13–31^, and few number of heterostructures of transition metal dichalcogenides^32–34^. The commonly used method to fabricate MSLs through stacking together mechanically exfoliated monolayers suffers from issues of uncontrolled stacking patterns and registry, contamination at the interfaces, and sample uniformity/size limitations^16,35–38^. Epitaxial growth approaches (e.g., based on chemical vapor deposition) can in principle overcome these problems, but are not yet widely reported^26,32,34^. The lack of diverse set of fabricated MSL materials impedes understanding of properties of these materials as well as applications. It is therefore desirable to find ways of fabricating the MSLs consisting of alternative 2D material sublayers and exploring their emergent unique properties.

Here, we report successful synthetic realization of large-scale homogeneous MSL based on a wide-gap 2D semiconductor, bismuth oxychloride (BiOCl), by using a facile and reproducible one-pot solvothermal approach. Bismuth oxychloride (BiOCl), which is a promising layered semiconductor, has received great attention owing to its optical and electrical properties that show potential for photocatalysis applications, such as water splitting^39^, dye degradation^40^, N_2_ reduction^41^, etc. Various nanocrystalline forms, including 2D nanosheets, have been synthesized by hydrolysis processes, hydrothermal/solvothermal and ionic-assisted methods^42^ with the aim of enhancing photocatalytic activity. The key for achieving the BiOCl MSL is bottom-up chemical growth of spiral-type nanosheets with 1.6–3.0° twist angles among adjacent sheets. The average size of spiral nanosheets is on the scale of 1.2 μm. Remarkably, the MSL exhibits a considerable band gap reduction of about 0.60 eV compared with bulk BiOCl, rendering BiOCl an optically active material in visible-light region. This is in comparison with homogeneous graphene-based MSLs which have shown band gap modulations below 0.05 eV^14^. Furthermore we find a more than twofold increase in carrier lifetime, which facilitates carriers separation for optoelectronics. As the result the nanosheets show much enhanced photocatalytic activity by comparison with bulk BiOCl. The experimental observations are supported by first-principles calculations, which ascribe the underlying mechanism to the locally enhanced interlayer coupling associated with the Moiré pattern, leading to a spatially modulated electronic structure feature. Our results, including demonstration of enhanced chemical functionality in an MSL, show the promise of exploiting the MSLs to modulate properties of 2D materials for practical electronic, optoelectronic and photocatalytic applications.

## Results

### Synthesis of spiral-type BiOCl nanosheets

**Fig. 1:**
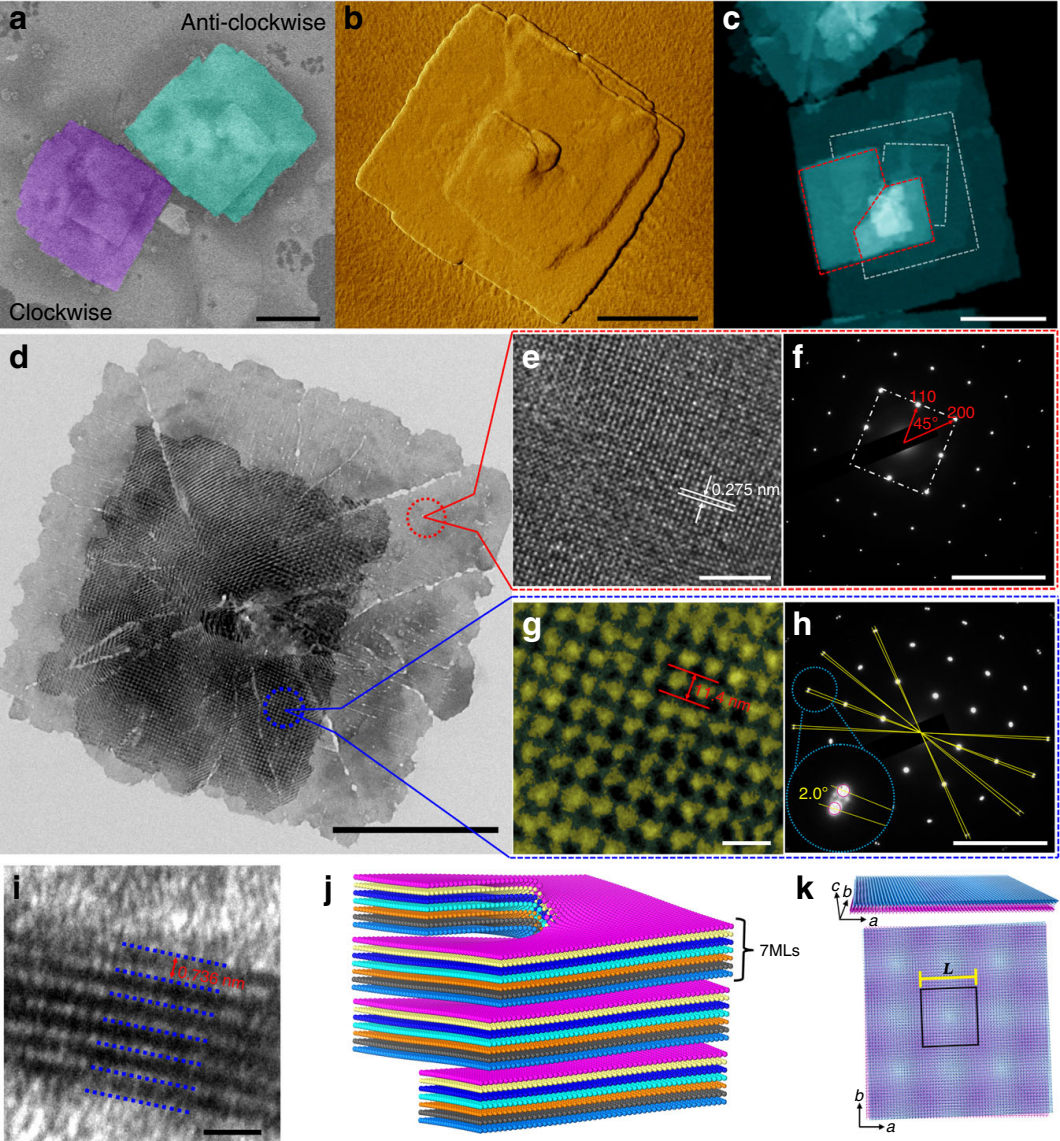
Morphological and structural characterization of BiOCl spiral nanosheets. **a** SEM images of BiOCl nanosheet with the spiral shape (either clockwise or anti-clockwise), scale bar: 500 nm. **b** AFM height-sensor micrograph of the nanosheet (scale bar: 500 nm). **c** HAADF-STEM image, scale bar: 500 nm. One can see two sets of distributed centrosymmetric screw related fringes (marked in red and white). **d** TEM image (scale bar: 500 nm). The cracks and irregular edges may be associated with the damage caused by the external forces during ultrasonic treatment and stirring in the process of sample preparation, or the result of relaxation of residual strains possibly existing in the nanosheets. Selected marginal region (in red circle) is measured by high-resolution TEM (**e**) (scale bar: 5 nm), and selected centered spiral region (in blue) is zoomed in to clearly show periodicity of Moiré pattern (**g**) (scale bar: 20 nm). **f**, **h** Selected area electron diffraction measurements for the regions of (**e**) and (**g**), respectively (scale bar: 10 1/nm). In (**h**), two sets of diffraction spots and formed intersection angle of 2.0° indicates adjacent nanosheets are twisted by 2.0°. **i** High-resolution TEM cross-sectional image of single nanosheet, which indicates each sheet consists of seven BiOCl atomic monolayers (scale bar: 2 nm). **j** Schematic of the grown BiOCl spiral nanosheets driven by the screw-dislocation. Each nanosheet consists of seven BiOCl monolayers (only O atoms are shown, in different colors). **k** 3D scheme and 2D perpendicular view of the bilayer Moiré superlattice model used in first-principles calculations. To clearly demonstrate Moiré pattern, only O atoms are shown and the upper and lower layers are shown in dodgerblue and purple, respectively. The period of Moiré superlattice *L* is indicated

The BiOCl nanosheets are synthesized via the solvothermal reaction by using easily hydrolyzed Bi(NO_3_)_3_·5H_2_O and poly diallyldimethylammonium chloride (PDDA) as precursors of Bi^3+^ and Cl^−^, respectively, and ethylene glycol as the solvent. Scanning electron microscopy (SEM) images (Fig. 1a) show that the produced nanosheets are tetragonal with an average side length of 1.2 ± 0.2 µm. The spiral shape is either clockwise or anti-clockwise. Atomic force microscopy (AFM) measurements on individual nanosheets (Fig. 1b and Supplementary Fig. 1a, b) suggest that the growth of spiral nanosheets was driven by screw-dislocation. The step height of the image indicates that each sheet has a thickness of 5.1 ± 0.3 nm. High-angle annular dark-field scanning transmission electron microscopy (HAADF-STEM) images (Fig. 1c) show two sets of distributed centrosymmetric screw related fringes (marked in red and white). This indicates that the spiral nanosheets are grown bidirectionally along the screw-dislocation axis. This bidirectional growth mode is further supported by the fact that the first step height of all the nanosheets from the AFM analysis is uniformly 10.3 nm (Supplementary Fig. 1b), which is twice of the thickness of an individual sheet. From the HAADF-STEM elemental mapping images (Supplementary Fig. 2a–d) demonstrating uniform distribution of Bi, O, and Cl throughout the entire sample and the X-ray diffraction results (Supplementary Fig. 2e), we conclude that the synthesized products are phase-pure BiOCl nanosheets. The clean nature of the BiOCl nanosheet surfaces is verified by the XPS analysis (Supplementary Fig. 3), where no observable signal in the N 1*s* spectrum is found. This indicates the absence of any residual reagent such as PDDA on nanosheet surfaces.

### Observation of Moiré superlattices

Figure 1d and Supplementary Fig. 4 show the transmission electron microscopy (TEM) image of the spiral nanosheet. Distinct from the marginal region (circled in red) showing a normal TEM diffraction pattern of BiOCl crystalline structure, the centered spiral region (circled in blue) exhibits squared Moiré patterns with a period *L* of 11.4 nm (Fig. 1g). The 2D BiOCl nanosheets are grown layer-by-layer along the [001] direction. The lattice space of 0.275 nm observed in the TEM image of Fig. 1e corresponds to the inter-plane distance among the {110} crystal planes. The diffraction spots of the selected area electron diffraction (SAED) measurement (Fig. 1f) agree with the in-plane tetragonal BiOCl lattice (with side and diagonal directions corresponding to {110} and {200} planes)^43^. The SAED pattern of the spiral nanosheet region (Fig. 1h) clearly shows two sets of diffraction spots with an intersection angle of ~2.0°. It suggests that the adjacent sheets are twisted by the angle of this magnitude. This resembles the cases of graphene or graphene/BN MSLs where the twisted bilayers lead to formation of Moiré patterns^4–6,13,22,24,28^. The period *L* of such MSLs is given by *L* = *a*_0_/sin*θ* where *a*_0_ is the length of in-plane Bravais lattice (3.89 Å for BiOCl)^43^ and *θ* is the twist angle. The calculated *L* in terms of this relation is 11.1 nm, in agreement with 11.4 nm measured directly from the TEM image (Fig. 1g). By measuring different synthesized spiral BiOCl nanosheets, we find the period *L* varies from 7.7 to 14.3 nm, corresponding to the twist angle ranging from 3.0° to 1.6° (Supplementary Figs. 5–6). In some spiral nanosheets with a uniform twist angle we occasionally observe different profiles of Moiré pattern in different regions (Supplementary Fig. 7). This can be ascribed to the slight deviation from flatness occurring in these nanosheets. The explanation is supported by the TEM results for the inclined samples (Supplementary Fig. 8), where the Moiré pattern changes profile with varying inclined angle. High-resolution TEM cross-sectional images (Fig. 1i) of the spiral nanosheets indicate that each sheet consists of seven BiOCl monolayers (in atomic sequence of [Cl–Bi–O–Bi–Cl]) with a 0.736 nm interval distance. This is consistent with the 5.1 ± 0.3 nm thickness of each sheet observed from the above AFM measurement (Fig. 1b). In some particular nanosheets, the side-view HRTEM image (where the cross section of the MSL was obtained by using the focused ion beam, Supplementary Fig. 9) indicates the sheet consists of nine BiOCl monolayers.

Therefore, the synthesized BiOCl MSLs are formed by the stacking of two nanosheets composed of multiple BiOCl monolayers with twist angle. Figure 1j shows a schematic of our spiral BiOCl nanosheet produced from the screw-dislocation driven growth. The incommensurate structure occurs between the bottom monolayer of the upper nanosheet and the top monolayer of the lower nanosheet. The distance between the two adjacent nanosheets is evaluated from the AFM step height image (Supplementary Fig. 1) and TEM measurement (Fig. 1i) as ~0.736 nm. The side-view HRTEM image (Supplementary Fig. 9) further indicates that the distance between the two adjacent nanosheets is equal to the length of *c*-axis for BiOCl lattice. In analogy to the usual MSLs formed by bilayers^3–9^, such incommensurately stacking between two nanosheets with multiple monolayers indeed leads to formation of MSLs (Supplementary Fig. 10). The key of the MSL is spatial in-plane modulation of atomic-scale structures, which leads to a spatial modulation of electronic structure and related properties. Such spatial modulation is directly related to the two outermost BiOCl monolayers being stacked with a twist angle. We thus adopt the BiOCl bilayers to represent the bottom layer of the upper sheet and the top layer of the lower sheet as shown in Fig. 1k. This captures the key structural features that are responsible for the spatial modulation of physical properties of interest.

### Morphologic characterization of grown Moiré superlattices

**Fig. 2:**
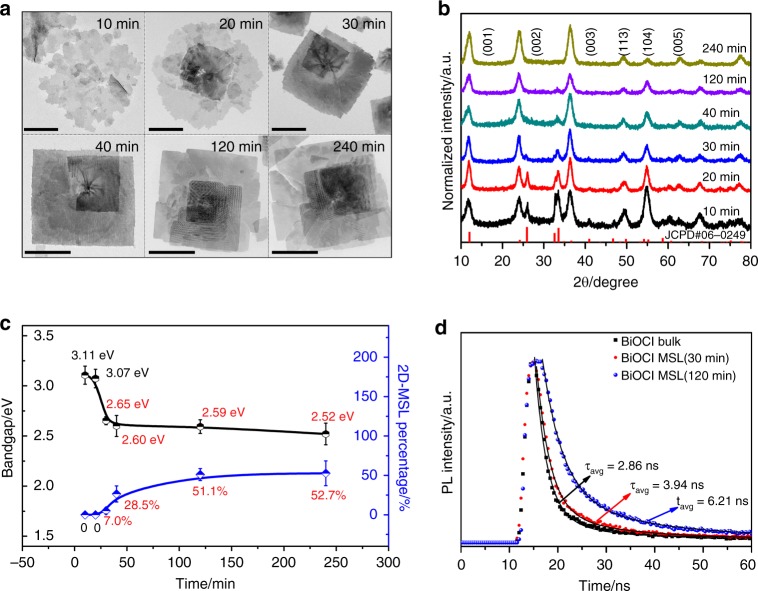
Evolution of BiOCl MSLs growth and different reaction times properties characterization. **a** TEM images and **b** XRD patterns measured at different reaction times during the BiOCl MSLs growth process (scale bar: 500 nm). In (**b**), red lines represent the benchmark data from the JCPDS card (#06-0249, BiOCl in space group P4/nmm). **c** Band gap values (in black) and existence percentage (in blue) of MSLs as the function of reaction time. Band gaps are deduced from absorption spectra (Supplementary Fig. 13). MSL percentage is evaluated based on the coverage of the spiral nanosheet region on the TEM images. The error bar represents the variation range of the data from multiple times of measurements. **d** Time-resolved photoluminescence spectra of BiOCl bulk (in black), BiOCl MSL nanosheets measured at the reaction time of 30 min (in red), 120 min (in blue), respectively. The measurements were taken via time-correlated single-photon counting (monitored at 455 nm) with an excitation wavelength of 365 nm. The average carrier lifetimes inferred from the emission decay (see main text) are indicated

To trace the evolution of the BiOCl MSLs growth, we measure snap-shot TEM images at different reaction times, as shown in Fig. 2a. A large incipient nanosheets with a slip plane is seen initially. After 20 min, the square sheet and gets thicker surrounding a central point are observed. After 30 min a well-defined spiral nanosheet is formed and centered at a screw-dislocation. Subsequently the size of the spiral nanosheet increases and one observes Moiré pattern away from the central screw-dislocation at about 40 min. The area of Moiré pattern shows substantial increase in size at 120 and 240 min. A schematic plot showing the procedure of formation of the spiral BiOCl nanosheets with interlayer twist angle is presented in Supplementary Fig. 11. The growth of spiral nanosheets with interlayer twist angle is attributed to the screw-dislocation driven self-perpetuating growth of spiral nanomaterials under the low supersaturation condition^44–48^, as described by the Burton-Cabrera-Frank model^49,50^. This shares the same mechanism with the “Eshelby twist” growth for 1D nanowires^51^. In our current case, we found that the spiral nanosheets of BiOCl cannot be obtained when the concentration of Bi(NO_3_)_3_ is higher than 0.4 mM and the concentration of PDDA higher than 2.25 wt% (Supplementary Fig. 12d).

The 2D spiral nanosheets grown with the screw-dislocation driven mechanism have been reported in Au^47^, transition metal dichalcogenides^52^, bimetallic hydroxides^53^, layered double hydroxides^54^, metal diboride^55^, metavanadate^56^, etc. But we observe Moiré patterns in the current BiOCl spiral nanosheets. The origin may be ascribed to the fact that our synthesized BiOCl nanosheets have high-quality, atomically thin, and large-area flat features and also suitable distance between the upper and lower sheets, favorable for the formation of MSL. The XRD data taken at the same time intervals (Fig. 2b) show persistent strengthening of (001), (002), and (003) peaks, indicating uniaxial-oriented growth of BiOCl MSLs. Some peaks missing in the XRD spectra for 40 min and above is ascribed to the broken in-plane crystal periodicity with the emergence of Moiré pattern in the incommensurately twisted stacking of the nanosheets.

### Emergent optoelectronic properties with Moiré superlattices

We then measure light absorption spectra of the spiral nanosheets at different stages and deduce band gap values from the standard Tauc plots (Supplementary Fig. 13). Figure 2c shows the band gaps (in black) and the percentages of Moiré pattern coverage (in blue) as a function of reaction time. The band gap of the BiOCl spiral nanosheet decreases from the original 3.11 to 2.65 eV after 30 min where only 7% coverage percent of MSL forms. The gap continuously decreases to a nearly constant value (~2.6 eV) when the MSL percentage approaches the coverage limit of ~50%. The ultraviolet photoelectron spectra (UPS) of the samples at 10 and 30 min (Supplementary Fig. 14) indicate a 0.42 eV up-shift of the valence band maximum (VBM), which is close to the 0.46 eV gap change. It is therefore the up-shift of VBM upon emergence of MSL that is responsible for the remarkable band gap reduction. Low-temperature EPR measurements of BiOCl nanosheets at different reaction times (Supplementary Fig. 15) show no observable signal. This excludes the role of easily formed oxygen vacancies as the origin of the change in optoelectronic properties. We note that in spite of no EPR signal revealing the existence of defects, there appears an observable trailing behavior in the visible region of UV/vis absorption spectra (Supplementary Fig. 13). This may ascribe to the emergent defects caused by the sample surface adsorbed hydroxyl and the laser irradiation during UV/vis spectra measurement^57^. The emergence of such defects may lead to band gap reduction to some extent, in addition to the predominated contribution from the formation of MSL as discussed. For comparison we have also synthesized a BiOCl bulk sample with similar size^58^ (Supplementary Fig. 16). This shows a similar band gap with our nanosheets at the initial growth stage before formation of the MSL.

Time-resolved photoluminescence (TRPL) measurements are performed to probe carrier dynamics of BiOCl MSLs, in comparison with BiOCl bulk samples (Fig. 2d and Supplementary Table 1). The emission decay data of BiOCl can be fitted by a biexponential function of *I*(*t*) = *B*_1_e^*−t*^/*τ*_1_ + *B*_2_e^*−t*^/*τ*_2_, where *I*(*t*) is the decay of fluorescence intensity at time *t*; *B*_1_, and *B*_2_ are the preexponential factors. The fast-process *τ*_1_ relates to nonradiative decay, whereas the slow-process *τ*_2_ comes from radiative recombination of photogenerated carriers^59^. The average carrier lifetime *τ*_avg_ is further evaluated by *τ*_avg_ = *A*_1_*τ*_1_ + *A*_2_*τ*_2_ with component proportion magnitudes *A*_1_ *=* *B*_1_*τ*_1_/(*B*_*1*_*τ*_1_ + *B*_2_*τ*_2_) and *A*_2_ *=* *B*_2_*τ*_2_/(*B*_1_*τ*_1_ + *B*_2_*τ*_2_). We find the high proportion of short component *A*_1_ for all the samples measured (>95%), consistent with the indirect band gap of BiOCl. With formation of the MSL, the photo-induced carriers decay more slowly. Specifically, we find that the average lifetime *τ*_avg_ changes from 2.86 ns in the bulk sample, to 3.94 ns in the spiral nanosheet at 30 min, and to 6.21 ns in the spiral nanosheet at 120 min. This suggests charge separation of photogenerated carriers in the MSLs, discussed further below.

### First-principles electronic structure calculations

**Fig. 3:**
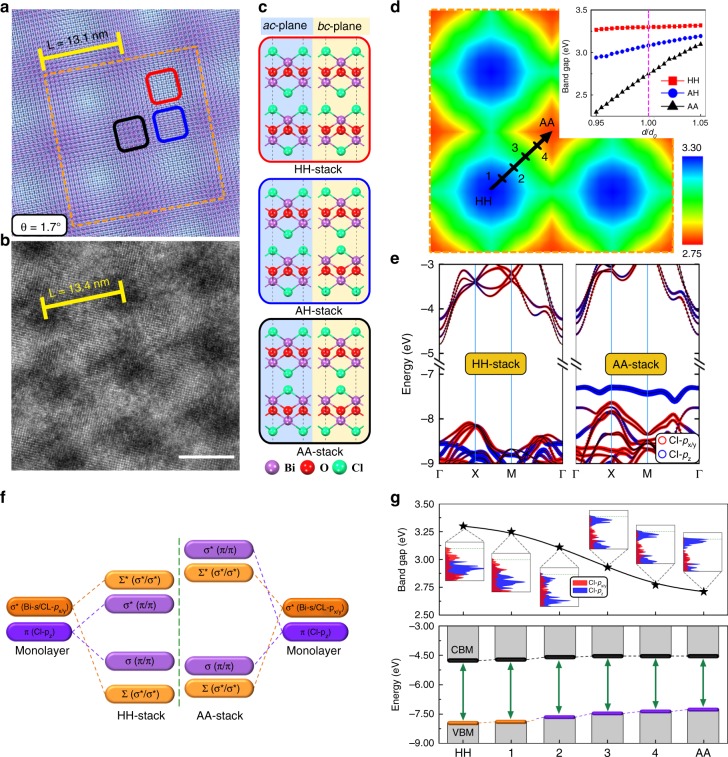
Electronic structure calculations of BiOCl MSL. **a** Perpendicular view of the simulated BiOCl spiral bilayer structure with the twist angle of 1.7°, forming a MSL with the periodicity *L* = 13.1 nm. To clearly demonstrate Moiré pattern, the upper and downer layers are shown in dodgerblue and purple, respectively. **b** HRTEM image of an experimentally synthesized BiOCl spiral nanosheet with the twist angle of ~1.7°, scale bar: 10 nm. **c** The bilayer structures with different stacking patterns, which dominate three distinct regions observed in (**a**) (indicated by red, blue, and black squares), named HH-stack, AH-stack, AA-stack structures, respectively. **d** Variation of calculated band gaps throughout the MSL, **E**_**g**_(**r**), shown within the yellow squared region of (**a**). The inset shows the dependence of band gap on the interlayer distance *d* for the HH-stack, AH-stack, AA-stack structures. *d*_0_ = 0.736 nm represents the experimentally determined distance between the two adjacent nanosheets. **e** Distinct band structures of the HH-stack and AA-stack structures. The band energies are aligned with respect to the vacuum energy level. The contributions from Cl-*p*_x/y_ and Cl-*p*_*z*_ orbitals are depicted by the sizes of red and blue circles, respectively. **f** Schematic of how the valence bands of HH-stack and AA-stack bilayers are formed by the states of composing monolayers. **g** Variation of band gap (upper panel) and band-edge positions (lower panel) evolving from the HH-stack to the AA-stack regions [i.e., the path indicated by the black arrow in (**d**)]. In the upper panel the Cl-orbitals projected density of states for each point is shown, and in the lower panel the chemical bonding character [as described in (**f**)] of the VBM state is given

We perform first-principles calculations to extract information about the physical origin of the emergent optoelectronic properties upon appearance of MSLs. A BiOCl spiral bilayer structure with the 1.7° twist angle is constructed, as shown in the perpendicular view of Fig. 3a. This leads to formation of the MSL patches distributed periodically with a period of 13.1 nm. The squared Moiré patterns with the period *L* from 7.7 to 14.3 nm and the corresponding twist angle from 3.0° to 1.6°, constructed using the bilayer MSL model, are given in Supplementary Fig. 17. Structurally they are similar apart from different periodicities. The square MSL pattern with one fourfold rotation and two mirror-planes symmetries clearly shows a periodic distribution of three distinct regions (labeled by red, blue, and black squares). The periodicity of the constructed MSL agrees well with the HRTEM image of the experimentally synthesized BiOCl spiral nanosheet with the nearly same twist angle (Fig. 3b). Analysis of crystal structures on the atomic scale indicates they are dominated by three bilayer structures with different stacking patterns (named HH-stack, AH-stack, AA-stack structures in Fig. 3c). The aberration-corrected HADDF-STEM analysis on single spiral BiOCl nanosheet (Supplementary Fig. 18) further confirms the experimental Moiré patterns involving three distinct regions of HH-stack, AH-stack, AA-stack structures (Supplementary Fig. 18c), in accordance with the structures from our simulation (Supplementary Fig. 18d). The nanosheet edge region characterized by the aberration-corrected HADDF-STEM (Supplementary Fig. 19) suggests the existence of high-indexed facets, which is indicated by the appearance of high density of low-coordinate (003) atomic steps, and few cases of (101) atomic steps. Among them the HH-stack is the ground-state stacking pattern of bulk BiOCl, whereas the other two are metastable higher-energy structures. We calculate the spatially resolved band gaps of MSL (E_g_(**r**)) by taking the local atomic structure (i.e., the particular stacking pattern at the position **r**). The interlayer distance of 0.736 nm, which is equal to the experimentally determined distance between the two adjacent nanosheets, is used. The detailed calculation procedure is described in Supplementary Note 16 (Supplementary Figs. 20–22) and the results are shown in Fig. 3d. One observes that the band gap E_g_(**r**) shows strong dependence on position **r**, varying from the minimum 2.75 eV in the AA-stack region to the maximum 3.30 eV in the HH-stack region. We note that the HH-stack pattern represents the ground-state BiOCl structure without formation of MSL. Therefore our calculations give a 0.55 eV band gap reduction of BiOCl upon emergence of MSL structure. This agrees with the value of ~0.6 eV from the absorption spectrum measurements (Fig. 2c).

### Origin of band gap reduction

Figure 3e shows a comparison of band structures of the HH-stack and AA-stack bilayers with the same interlayer distance of 0.736 nm. The band gap reduction in the AA-stack structure is clearly caused by the up-shift of valence bands. This is consistent with the experimental UPS analysis (Supplementary Fig. 14). Atomic orbital projections of band structures show that while the VBM of the HH-stack structure comes mainly from Cl-*p*_x/y_ orbitals, the VBM of the AA-stack structure is derived from the Cl-*p*_z_ orbital. Further analysis (Fig. 3f) indicates that when two monolayers are stacked to form bilayers, for the HH-stack structure the VBM originates from the weak anti-bonding interaction between two monolayer anti-bonding σ*(Bi-*s*/Cl-*p*_x/y_) states, ∑*(σ*/σ*), whereas for the AA-stack structure the VBM is made up of the anti-bonding combinations of the π(Cl-*p*_z_) states of the two monolayers, σ*(π/π). The distinct valence band formation of the AA-stack structure originates from its crystal structure with the head-to-head arrangement of the Cl atoms from adjacent layers (lower panel of Fig. 3c). This remarkably enhances the interaction between the adjacent monolayer π(Cl-*p*_z_) states, raises the energy levels of the anti-bonding σ*(π/π) states, and makes them become the valence band-edge states. As the result when the interlayer distance *d* varies, the AA-stack structure exhibits pronounced up-shift of valence bands and dramatic change in band gap (inset of Fig. 3d). The AH-stack is an intermediate structure between the AA-stack and HH-stack structures. It is therefore not surprising that the AH-stack structure demonstrates a modest band gap change with varying interlayer distance, falling between those of AA-stack and HH-stack structures. Within the whole BiOCl MSL, the AH-stack structure is expected to make its contribution to the total optical spectrum, but not determine the optical threshold. Figure 3g shows the evolution of band gaps, band-edge energies, and components of band-edge states with the structure change from the HH-stack to the AA-stack patterns. We clearly see that the band gap reduction can be ascribed to the up-shift of the VBM, accompanying with the corresponding change of its constituted monolayer states with individual atomic orbital features. Therefore the large band gap reduction is traced down to the AA-stack region of the MSL, which sustains the shorter interlayer distance than its equilibrium condition. Such shorter interlayer distance substantially enhances the interlayer coupling^60–62^, upshifts the valence band edge, and reduces the band gap.

### Origin of increased carrier lifetime

It is known that photo-excited electrons and holes of optoelectronic materials will tend to diffuse to the regions where the conduction band minimum (CBM) is lowest and the VBM is highest, respectively. From Fig. 3g (lower panel) we see that while the CBM decreases slightly from the AA-stack structure to the HH-stack structure, the VBM is clearly maximized at the AA-stack region. Thus electrons would tend to flow toward the HH-stack region, whereas holes would tend to flow to the AA-stack region and get localized there. This leads to photogenerated charge separation in BiOCl MSLs, which explains the observed slower carrier decay kinetics from the TRPL measurement (Fig. 2d).

**Fig. 4:**
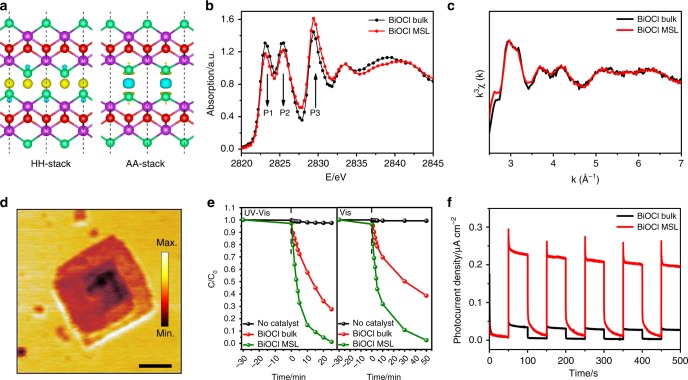
Charge carriers characterization and photocatalysis of BiOCl MSLs. **a** Charge density differences between the bilayers (in AA-stack) of BiOCl MSL and individual monolayers (in HH-stack) of BiOCl bulk. The isosurface value is 0.002 electrons/Å^3^ and the charge accumulation and depletion are denoted in yellow and blue, respectively. **b**, **c** Comparison of XANES (Cl K-edge) and EXAFS spectra collected on BiOCl bulk and BiOCl MSL samples, respectively. In (**b**) the pre-edge P1 and P2 peaks represent the transitions from Cl 1*s* orbital to unoccupied hybridized Bi 6*p* and Cl 3*p* orbitals, and the P3 peak represents the Cl 1*s* → Cl 4*p* transition^63^. **d** The near-field amplitude image from s-SNOM measurement showing distribution of free carriers. One observes that the centered MSL region shows the lower local carrier density indicated by the weaker near-field amplitude (scale bar: 500 nm). **e** Photocatalytic degradation rate of Rhodamine B versus time without catalyst and with BiOCl bulk, BiOCl MSL nanosheets as catalysts. Negative time denotes dark condition before light irradiation. The measurements are taken under simulated sunlight equipped with an AM 1.5 G filter (UV-Vis) and visible-light (Vis) irradiation, respectively. **f** Transient photocurrent response versus time without bias, measured in a 0.5 mol L^−1^ Na_2_SO_4_ aqueous solution and the Ag/AgCl reference electrode under visible-light irradiation

Figure 4a shows charge density differences of the HH-stack (left panel) and AA-stack (right panel) bilayers with respect to the monolayers. We see distinct chemical bonding between layers: the HH-stack structure shows modest amount of charge accumulation around Cl atoms in the interlayer region. This results from overlap of the Cl-*p*_x/y_ states of monolayer. In contrast, the AA-stack structure exhibits substantial charge depletion between head-to-head arranged Cl atoms from adjacent layers, which indicates a strong interlayer anti-bonding interaction. By comparison with BiOCl bulk phase, the emergence of distinct interlayer bonding associated with the AA-stack (and also AH-stack, see Fig. 3a–c) regions in the MSL is further supported by the X-ray absorption near-edge structure (XANES) spectrometry and extended X-ray absorption fine structure (EXAFS) results as shown in Fig. 4b, c. The EXAFS data of the MSL and bulk samples are nearly same (after excluding noise signals), indicating their common covalent bond lengths within the BiOCl layer. For the XANES results, the pre-edge P1 and P2 peaks, which represent the transitions from Cl 1*s* orbital to unoccupied hybridized Bi 6*p* and Cl 3*p* orbitals^63^, show clear differences between these two cases. This originates from their distinct bonding/interaction among layers that have essential effect on the unoccupied conduction bands formed by hybridized Bi 6*p* and Cl 3*p* orbitals. The Bi L_III_-edge XANES profiles (Supplementary Fig. 23a) for spiral nanosheet and bulk samples are nearly identical, consisting with the same chemical state of Bi in different phases. The Bi L_III_-edge EXAFS profiles (Supplementary Fig. 23b) indicate that the intensities of Bi–O peak and Bi–Cl peak of spiral nanosheet are slightly lower than those of bulk sample. This may be attributed to the emergent more diverse local atomic coordination environments in the spiral nanosheet associated with the more complex structure containing the screw-dislocation leading to formation of the Moiré pattern. We characterize the spatial distribution of majority carriers (these are n-type based on Mott–Schottky analysis shown in Supplementary Fig. 24) across the spiral nanosheet by using scattering-type scanning near-field optical microscopy (s-SNOM) as shown in Fig. 4d. Clearly the centered MSL region exhibits the lower local carrier density indicated by the weaker near-field amplitude. Since the measurement is taken under steady state conditions after sufficient carriers radiative/nonradiative recombination, the result gives indirect evidence of hole aggregation in the MSL region^64^. This is in accordance with the above analysis that the hole carriers would be localized at the AA-stack region of the MSL based on the bands alignment data (Fig. 3g).

Photo-induced electron and hole carriers are expected to have improved transfer with the emergence of the MSL. This is based on consideration of the two aspects: (i) Based on the calculated CBM change from the AA-stack to HH-stack bilayers (Fig. 3g), the electrons tend to flow from the AA-stack toward HH-stack regions in the MSL. (ii) The calculated VBM change from the AA-stack to HH-stack bilayers (Fig. 3g) indicates a hole localization tendency at the AA-stack region, which is also evidenced by the s-SNOM results (Fig. 4d). However, the VBMs vary gradually from one region to the other. The gradually varying energy of hole states, with the existing concentration gradient among different regions, would be expected to result in reasonable hole transfer out of the AA-stack region.

### BiOCl Moiré superlattices as superior phototcatalysts

We investigate the photocatalytic performance of the spiral nanosheets, finding emergent chemical functionality in the MSLs. Bulk BiOCl (Supplementary Fig. 16) is explored for comparison. The photocatalytic degradation of Rhodamine B with BiOCl as the catalyst is chosen for this purpose. The results are shown in Fig. 4e and Supplementary Fig. 25. The Rhodamine B (RhB) degradation is detected by the change in intensity of its characteristic peak at 553 nm in temporal UV-vis absorption spectrum (Supplementary Fig. 25). We find that with the MSL sample as catalyst, the RhB shows much more dramatic degradation than the case catalyzed by the bulk sample. Particularly, when the degradation efficiency over the MSL sample reaches 99% at 25 min under simulated AM 1.5 G solar irradiation (or 50 min under visible-light irradiation), the efficiency over the bulk sample reaches only 73% (and 62% under visible-light irradiation). The remarkably improved photocatalytic performance of BiOCl MSLs is attributed, on the one hand, to its reduced band gap from 3.11 eV of bulk phase to ~2.60 eV (Fig. 2c) that falls into the visible-light region, enhancing light capture efficiency, and on the other hand, to its photogenerated charge separation and resulting increased carrier lifetime—both consequences of the MSL formation. The latter is further evidenced by the transient photocurrent response measurement (Fig. 4f), where the MSL sample exhibits a five times higher photocurrent than the bulk sample. It should be pointed out that the defect structures (i.e., low-coordinated sites) around the screw-dislocation axis may also contribute to the promotion of photocatalytic activity of BiOCl MSLs. To exclude this effect we compare directly the visible-light photocatalytic activity data of the BiOCl MSLs (30 min) and BiOCl MSLs (120 min) (Supplementary Fig. 26), which have almost the same area of the central screw-dislocation region, but different coverage percent of the MSL. The results show substantially enhanced photocatalytic activity in the BiOCl MSLs (120 min). This further evidences that the enhancement of photocatalytic activity is attributed to the emergence of MSL inducing strong modulation of optoelectronic properties as discussed above.

As a photocatalyst, in addition to the improved performance, the BiOCl MSLs exhibit also good light stability. As shown in Supplementary Fig. 27, the photo-degradation rate remains constant over five consecutive cycles. This indicates its robust recycling stability under visible-light irradiation. To further confirm high photocatalytic performance of the BiOCl MSLs, we measured their visible-light photocatalytic activity in the photo-degradation process of colorless tetracycline (TC) antibiotic molecule (Supplementary Fig. 28). Compared with the bulk sample, the MSL clearly shows enhanced photocatalytic activity for degrading the colorless TC. Particularly, after 100 min visible-light illumination, about 83.8% TC is degraded by the MSL, while only 12.1% TC is degraded by the bulk. We note that the oriented axis, high-indexed facets, and edge sites may be the reaction sites on BiOCl MSLs during catalytic degradation, though the detection of explicit reaction sites is challenging and beyond the scope of this study.

## Discussion

In summary, we have demonstrated the bottom-up synthesis of 2D Moiré superlattices (MSLs) based on a wide-gap oxychloride semiconductor BiOCl. The controlled growth was conducted in facile solvothermal reaction condition, producing spiral-type nanosheets under a screw-dislocation driven mechanism. The homogeneous MSL based on one semiconductor is of high crystalline quality, free of contamination, robustly reproducible and scalable, and uniform sample distribution. More remarkably, the fabricated BiOCl MSLs leads to strong modulation of optoelectronic properties including up to 0.6 eV band gap reduction, substantial carrier lifetime increase, and resulted strongly enhanced visible-light photocatalytic activity. We exploit the idea of formation of 2D MSLs and provide a clean modification of optoelectronic properties in pristine BiOCl without introducing any external impurities/materials. This is distinct from the usual strategies for modifying the optoelectronic properties of BiOCl, such as doping with impurities, encapsulation involving metal nanoparticles, heterostructures with other semiconductors, etc. First-principles calculations reveal that it is the locally enhanced interlayer coupling upon appearance of the Moiré pattern that results in modification of electronic band structure and optoelectronic properties. By achieving such highly scalable MSLs with modulated photophysical properties, our work advocate the promise of 2D MSL family in optoelectronics, photocatalytic and practical environmental purifications. Finally, our synthesized MSLs are formed by the stacking of two spiraling nanosheets with one twist angle. It is possible to synthesize multiple spiraling nanosheets with the constant or changed twist angles between two adjacent sheets. The former will result in multiple MSLs stacked along the perpendicular direction with the same periodicity, whereas the latter will give rise to the MSLs with the changed periodicity. For such reassembly of the MSLs, the optical spectral properties will be contributed by individual MSLs, and there could be a gradient in properties such as bands edges, carrier concentration depending on the details.

## Methods

### Materials synthesis

The reactant materials to synthesize BiOCl spiral nanosheets, Bi(NO_3_)_3_·5H_2_O (99%) and ethylene glycol (EG, 99%) were purchased from Sinopharm Chemical Reagent Co. Ltd. (Shanghai, China). Poly(diallyldimethylammonium chloride) (PDDA, 20 wt% in water, MW = 400,000–500,000) and tetracycline (TC) were obtained from Aladdin. All chemicals were used as received without further purification. Ultrapure water (18.2 MΩ cm^−1^) was used in all the experiments. In a typical synthesis process of BiOCl spiral nanosheet, 4.8 ml (1.13 wt%) PDDA was added in 80 ml of EG at room temperature with continuous stirring, and then 0.0971 g (0.2 mM) Bi(NO_3_)_3_ 5H_2_O was dissolved in the above solution. The mixture was stirred for 30 min and further heated to 200 °C in a 250 ml two-neck flask equipped with a reflux condenser. After the temperature maintained at 200 °C for 2 h, and the mixture was cooled down to room temperature naturally. The white products were collected by centrifugation, washed with deionized water three times to remove residual ions, and finally dried at 60 °C overnight in vacuum. For comparison purpose the preparation of BiOCl bulk sample were carried out using the method reported^58^.

### Materials characterization

Powder X-ray diffraction patterns (XRD) were obtained by using a Bragg–Brentano diffractometer (D8-tools, Germany) with a Cu-Kα line at 0.15418 nm. Scanning electron microscopy (SEM) images were taken by using the Ultra-High Resolution (1.0 nm) Scanning Electron Microscope SU-8010 (HITACHI Co., Japan). The powders of BiOCl spiral nanosheet dispersed in water were dropped on 300 mesh carbon-coated copper grids and dried for the characterizations of transmission electron microscopy (TEM), high-resolution transmission electron microscopy (HRTEM) and selected area electron diffraction (SAED). These were acquired by using a JEM-2100F transmission electron microscope (JEOL Co., Japan). High-angle annular dark-field scanning transmission electron microscopy (HAADF-STEM) images and energy-disperse X-ray (EDX) elemental mapping were measured by using a FEI Tecnai G2 F20 transmission electron microscope equipped with a high-angle annular dark-field detector (FEI Company, USA). Atomic force microscopy (AFM) measurements were performed on the XE-100 multimode AFM from Park Systems (USA). Ultraviolet–visible diffused reflectance spectra were collected by using an ultraviolet–visible spectrophotometer (Shimadzu UV-2550, with BaSO_4_ as a reference) and were then converted from reflection to absorbance by the Kubelka-Munk method. The ultraviolet photoelectron spectra (UPS) measurements were carried out using an ESCALAB MK II instrument (Thermo Fisher Scientific, USA). The X-ray photoelectron spectroscopy (XPS) was obtained over Thermos ESCALAB 250 spectrometer, using a monochromatized Al Kα radiation. Electron paramagnetic resonance (EPR) spectra were recorded at 123 K using EPR spectrometer (A300-10/12, Bruker, Rheinstetten, Germany). Aberration-corrected scanning transmission electron microscopy (STEM) images of BiOCl MSL were obtained by a JEOL ARM200F (JEOL, Tokyo, Japan) operated at 200 kV with cold field emission gun and double hexapole Cs correctors (CEOS GmbH, Heidelberg, Germany). The fluorescence decay processes were recorded with the time-correlated single-photon counting (TCSPC) technique on an Edinburgh FLS920 phosphorescence lifetime system equipped with a 450 W Xe lamp and a time-correlated single-photon counting card at room temperature. Nanoscale mapping of free carriers in the spiral nanosheets were performed by scattering-type scanning near-field optical microscopy (s-SNOM). The s-SNOM system is built based on an atomic force microscope (AFM) that is illuminated by a focused infrared (IR) laser beam. Free-carrier mapping is achieved by recording the amplitude and phase of light backscattered from the metalized AFM tip, simultaneously to the topography. X-ray absorption spectra (XANES) were collected at Beijing Synchrotron Facility (BSRF) on beamline 4B7B. The BSRF storage ring is operated at the electron energy of 2.2 GeV with beam current of 250 mA. A Si (111) double crystal monochromatic was applied. The beam size used at the sample position is about 900 × 300 μm^2^. All the data were collected with the transmission mode at ambient temperature. Curve fitting and data analysis were performed with Artemis and IFEFFIT software.

### Photocatalytic activity measurements

Rhodamine B (RhB) for photocatalytic degradation were purchased from Sinopharm Chemical Reagent Co. Ltd. (Shanghai, China). The photocatalytic activity of the BiOCl MSL and bulk samples has been evaluated for photodecomposition of RhB under simulated sunlight from a 300 W Xenon lamp equipped with an AM 1.5 G filter (UV-Vis) or a 400 nm cutoff filter (Vis), respectively. The reaction temperature was controlled to be 24 °C by a thermostat bath. Typically 0.02 g photocatalyst was dispersed in 50 mL RhB solution (10 mg L^−1^) in a reaction cell with a Pyrex jacket by sonication for 5 min. Prior to irradiation, the suspension was stirred in the dark for 30 min for adsorption–desorption equilibrium. Three milliliters of the suspensions were withdrawn and centrifuged (12,000 × *g*, 10 min) to separate the photocatalyst for UV-Vis spectrophotometer measurements (Shimadzu UV-2550). The concentration of RhB was determined by monitoring its characteristic absorption peak at 553 nm from UV-vis absorption spectra. The degradation efficiency (*E*) of RhB was calculated by the formula of $E = \frac{{C_0 - C}}{{C_0}} \times {\mathrm{100\% }}$, where *C*_0_ and *C* are the initial concentration (10 mg L^−1^) and the instant degradation concentration of Rhodamine B during the degradation process, respectively.

The photocatalytic tetracycline (TC) solution (20 mg L^−1^) measurements are implemented at ambient temperature (24 °C). Fifteen milligrams of catalyst is dispersed in 50 ml TC solution by sonication. Then, the reactant mixture is continously stirred in the dark for 30 min, then illuminated by visible light (*λ* > 400 nm). A two milliliters suspension were withdrawn and centrifuged during the reaction. Finally, the concentration variation of TC was examined by a UV-Vis spectrophotometer.

### Photoelectrochemical measurements

The photocatalyst powder was dispersed in H_2_O to form a 10 mg mL^−1^ solution under sonication for 30 min. Indium-tin oxide (ITO) glass, as the working electrode, was cleaned by sonication in cleanout fluid, acetone and ethanol for 10 min. The as-prepared solution was dropped onto the pretreated ITO surface and allowed to dry under vacuum conditions for 24 h at 60 °C. Subsequently, the uncoated part of the ITO glass was isolated with epoxy resin. The photocurrent was measured by an electrochemical analyzer (CHI660D Instruments) in a conventional three-electrode electrochemical cell with the working electrode, a platinum foil counter electrode and Ag/AgCl (3 M KCl) as reference electrode. A 300 W Xenon lamp with 400 nm cutoff filter was utilized as a light source. A 0.5 M Na_2_SO_4_ aqueous solution was used as the electrolyte. The Mott–Schottky measurements were carried out using a Bio-Logic SP-150 potentiostat equipped with a VMP3B-20 20A booster.

### First-principles electronic structure calculations

First-principles calculations were carried out within the framework of density functional theory (DFT) by using plane-wave pseudopotential methods as implemented in the Vienna Ab initio Simulation Package^65,66^. We described the electron-ion interactions by using the projected augmented wave pseudopotentials with the 6*s*^2^6*p*^3^ (Bi), 3*s*^2^3*p*^5^ (Cl), 2*s*^2^2*p*^4^ (O) treated explicitly as valence electrons. We used the generalized gradient approximation formulated by Perdew, Burke, and Ernzerhof^67^ as exchange correlation functional. The layered BiOCl system was embedded in a vacuum region with 30 Å thickness. A kinetic energy cutoff of 520 eV was used for wave-function expansion, and a 11 × 11 × 1 *k*-point grid within the Monkhorst-Pack scheme was adopted for electronic Brillouin zone integration. To consider the dispersive van der Waals interaction that cannot be ignored in the current layered system, the optB86b-vdW functional^68^ was used and was found to give the equilibrium lattice parameters (*a* = 3.89 Å, *c* = 7.36 Å) that agree well with experimental values (*a* = 3.89 Å, *c* = 7.37 Å)^43^. Structure optimizations (including cell parameters and internal atomic positions) were performed using the conjugate gradient technique, until the total energies converged to 10^−5^ eV. To simulate BiOCl Moiré superlattices, we constructed a BiOCl spiral bilayer structure with the 1.7° twist angle at the interlayer distance of 0.736 nm (from TEM and AFM measurements). The interlayer distance was fixed during the structure optimization. To remedy the band gap underestimation in DFT based calculations, we employed the hybrid functional (HSE)^69^ approach (with 21.5% exact Fock exchange), which gives a 3.45 eV band gap for BiOCl bulk phase, showing agreement with the experimental 3.17–3.40 eV^58,70^. Spin-orbit coupling (SOC) is included in the electronic structure calculations.

## Supplementary information

Supplementary Information

Peer Review

Source Data

## Data Availability

The data underlying Figs. 2–4, Supplementary Figs. 1, 3, 6, 13–15, 23, and 25–28 are provided as a Source Data file. The other data support the findings of this study are available from the corresponding author upon request.
