# Supplementary material for: Bottom-up growth of homogeneous Moiré superlattices in bismuth oxychloride spiral nanosheets
Source: Nat Commun. 2019 Oct 2;10:4472. doi: 10.1038/s41467-019-12347-7 (PMC6775108; doi:10.1038/s41467-019-12347-7)
Supplement: Supplementary file 1 — Supplementary Information [file 41467_2019_12347_MOESM1_ESM.pdf]

Supplementary Information

Bottom-up growth of homogeneous Moiré superlattices in bismuth oxychloride spiral nanosheets

Liu et al.

Supplementary Note 1

Atomic force microscopy (AFM) measurement is performed to characterize the morphology of an individual BiOCl nanosheet, as shown in the AFM height-sensor micrograph of Supplementary Figure 1a. The step height profile along the dash arrow line is shown in Supplementary Figure 1b. Two conclusions can be drawn from the measured data: (i) Each sheet has a thickness of 5.1 ± 0.3 nm; (ii) The first step height of the nanosheet is 10.3 nm, twice of the thickness of each sheet. This indicates a screw-dislocation driven bidirectional growth of spiral nanosheet.

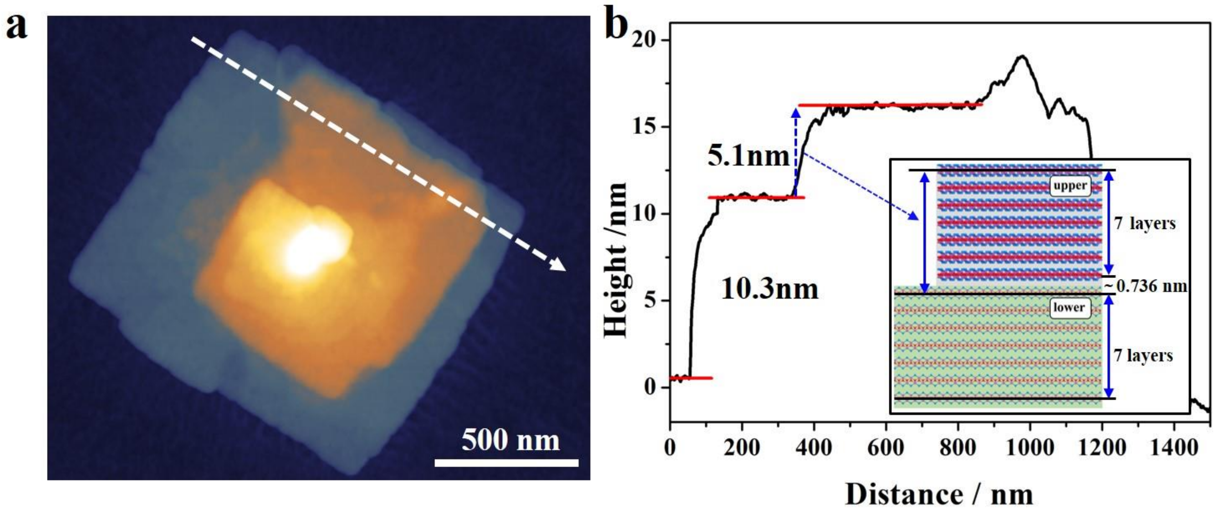

Supplementary Figure 1: a) AFM height-sensor micrograph of an individual BiOCl nanosheet. b) One-dimensional step height profile along the dash arrow line in (a). The inset indicates how the distance (~0.736 nm) between the two adjacent nanosheets is determined.

Supplementary Note 2

We perform elemental mapping measurement (Supplementary Figure 2a-2d) via high-angle annular dark-field scanning transmission electron microscopy (HAADF-STEM), and X-ray diffraction (Supplementary Figure 2e) to characterize crystalline structure of BiOCl nanosheet. Elemental mapping results show uniform distribution of Bi, O, and Cl throughout the entire sample. Excellent agreement between our measured XRD patterns and the JCPDS card data indicates that our synthesized samples are phase-pure BiOCl in the tetragonal P4/nmm structure. The sharper (001), (002) and (003) diffraction peaks with the stronger intensities among other peaks further indicate that the nanosheets are grown layer-by-layer orientated along the [001] direction (see the main text).

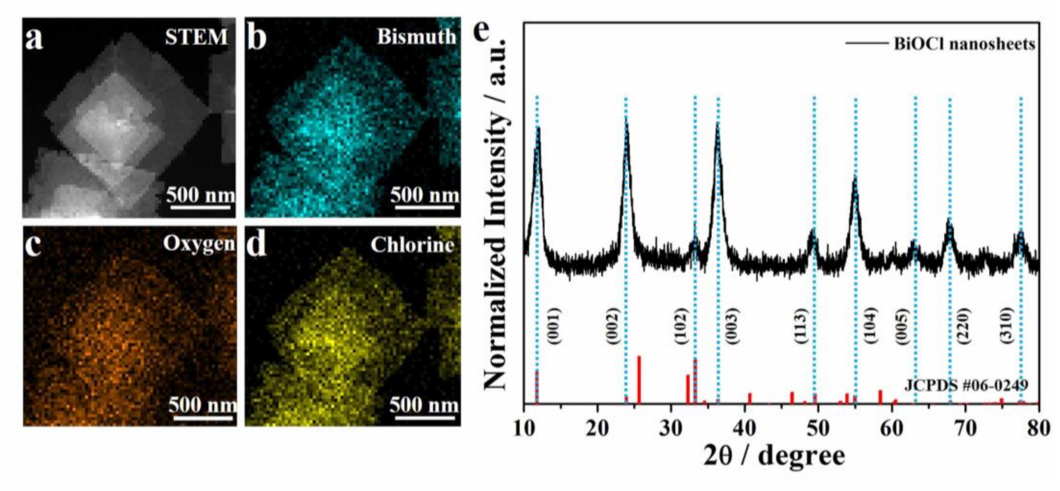

Supplementary Figure 2: a-d) HAADF-STEM image of BiOCl nanosheet and elemental mapping measurements showing distribution profiles of Bi (blue), O (orange), and Cl (yellow) elements. e) Measured XRD patterns of the nanosheet. The benchmark data from the JCPDS card (no. 06-0249, BiOCl in space group P4/nmm) is shown for comparison.

Supplementary Note 3

X-ray photoelectron spectroscopy (XPS) analysis is performed to investigate whether the residual reagent exists on the surface of BiOCl nanosheets. The N 1*s* spectrum in the Supplementary Figure 3 do no show observable signals that are related to residual reagent such as PDDA.

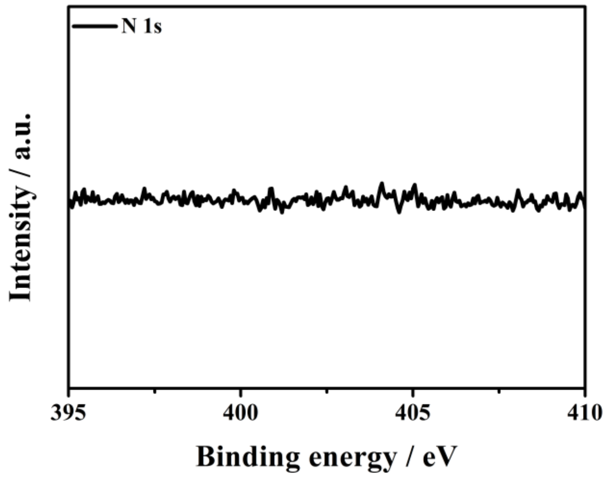

Supplementary Figure 3: N 1*s* XPS spectrum of BiOCl MSL nanosheets.

Supplementary Note 4

To further prove the monodispersity of BiOCl MSL, we add the low-magnification TEM image showing more our synthesized nanosheets as in the Supplementary Figure 4. All the nanosheets show the visible Moiré patterns, which are caused by the small degree of interlayer twist angles between two nanosheets.

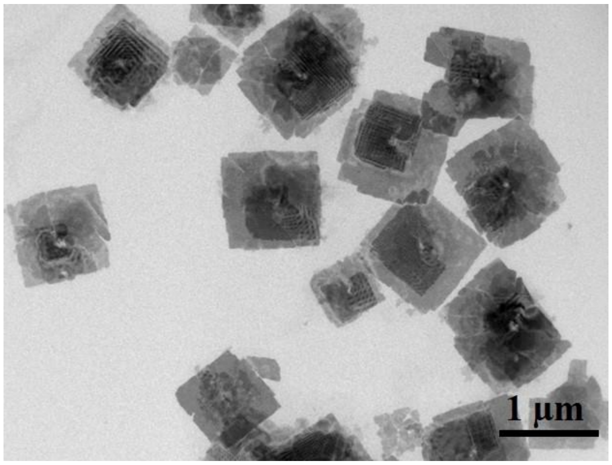

Supplementary Figure 4: Low-magnification TEM image of BiOCl MSL nanosheets.

Supplementary Note 5

We find that different spiral BiOCl nanosheets have varying periodicities of Moiré pattern. As shown in the TEM image of a nanosheet (different from the one shown in Figure 1d of the main text) in Supplementary Figure 5a-b, the MSL region shows a periodicity of 13.4 nm. Following the relationship between the MSL periodicity L and the twist angle θ among adjacent sheets, $L = a_0/\sin\theta$ (a_0 is the length of in-plane Bravias lattice of BiOCl, 3.89 Å), the deduced twist angle is 1.65°. This is in accordance with the measured angle of 1.7° from the selected area electron diffraction (SAED) measurement (Supplementary Figure 5c).

We measure MSL periodicities of totally 30 spiral nanosheets (from their amplified TEM images). The statistic results are summarized in Supplementary Figure 6 which shows the appearance frequency of MSLs as the function of their periodicities. For each periodicity, the corresponding twist angle among adjacent sheets is also indicated. One sees that the periodicities vary from 7.7 to 14.3 nm, corresponding to the twist angles in range of 3.0°-1.6°. The majority of MSLs have their periodicities of 11.0-13.3 nm (corresponding to the twist angles of 2.1°-1.7°).

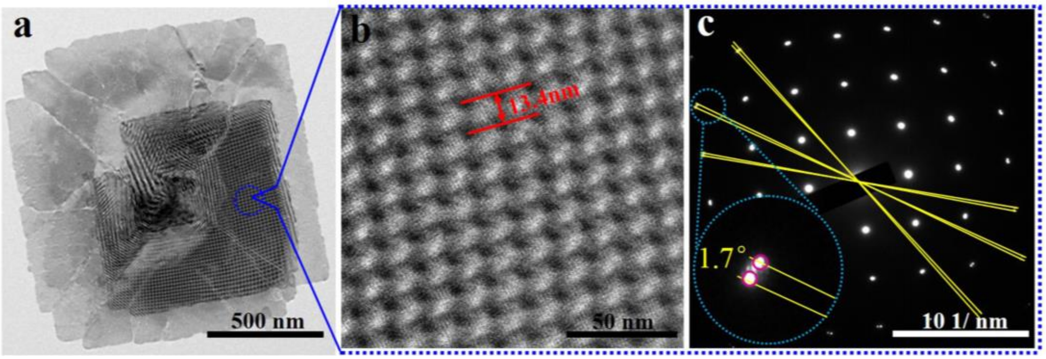

Supplementary Figure 5: a) TEM image of an individual spiral BiOCl nanosheet (different from the one shown in **Figure 1d** of the main text). b) Amplified TEM image of selected centered spiral region (in blue) of (a) to clearly show periodicity of Moiré pattern. c) SAED measurement of selected spiral region (in blue) of (a).

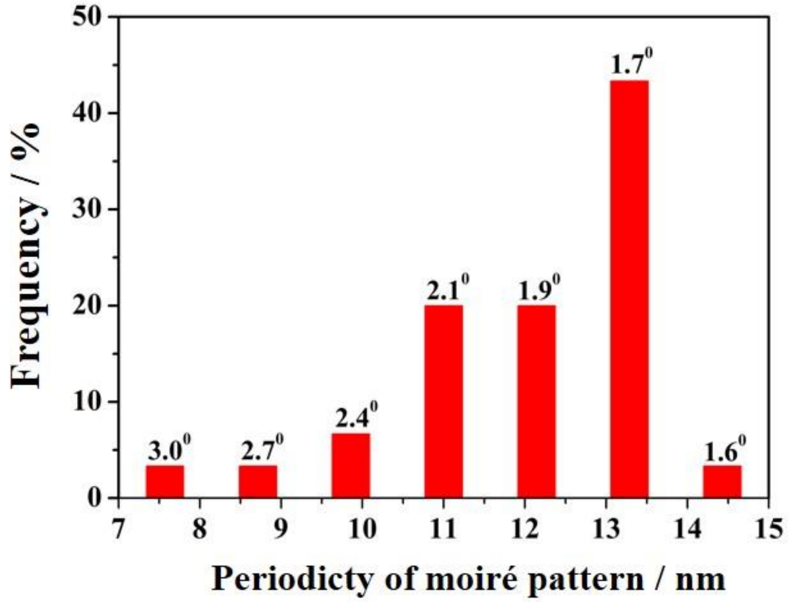

Supplementary Figure 6: Appearance frequency of BiOCl MSLs as the function of their periodicities. The statistic results are taken from the measurements of totally 30 spiral nanosheets. The twist angle corresponding to each periodicity is indicated.

Supplementary Note 6

We find that in one spiral BiOCl nanosheet with the exactly same twist angle (Supplementary Figure 7a and Supplementary Figure 7c), the moiré patterns at different regions may show different profiles (Supplementary Figure 7b). We attribute this behavior to the slight out-of-flatness nature of the nanosheet. This is indirectly evidenced by the TEM images of the same nanosheet measured under the conditions that the sheet is inclined by different angles (Supplementary Figure 8). One observes that in the same region of the nanosheet, the Moiré pattern changes profile with varying inclined angles. Similarly the out-of-flatness nature of the nanosheet can result in different moiré patterns in different regions.

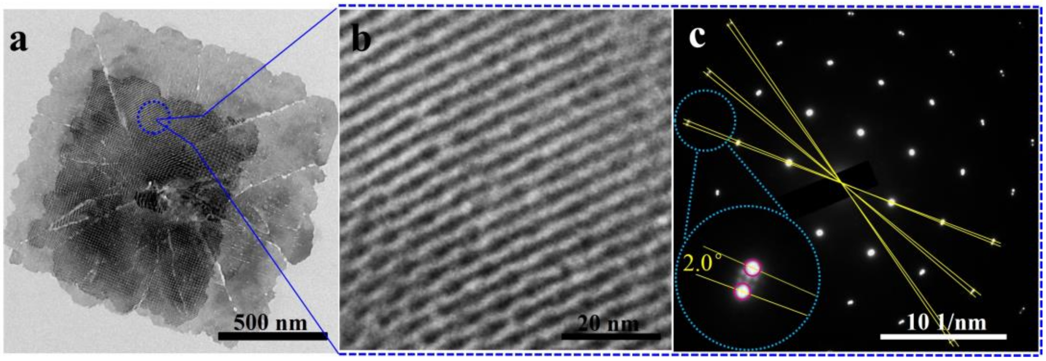

Supplementary Figure 7: a) TEM image of the same nanosheet shown in Fig. 1d of the main text. b) Amplified TEM image of selected centered spiral region (in blue) of (a) to clearly show profile of moiré pattern. c) SAED measurement of selected spiral region (in blue) of (a).

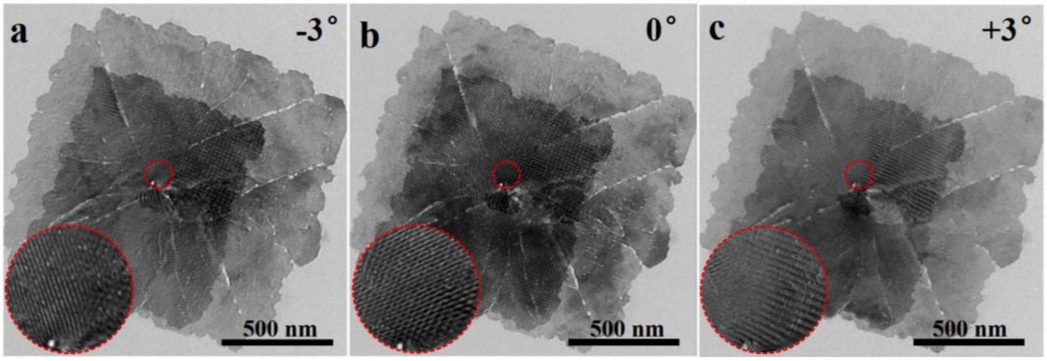

Supplementary Figure 8: TEM images of the same nanosheet measured under the conditions that the sheet is inclined by the angles of a) -3°, b) 0°, and c) +3°. One observes that in the same region of the nanosheet the moiré pattern change profile with the varying inclined angles.

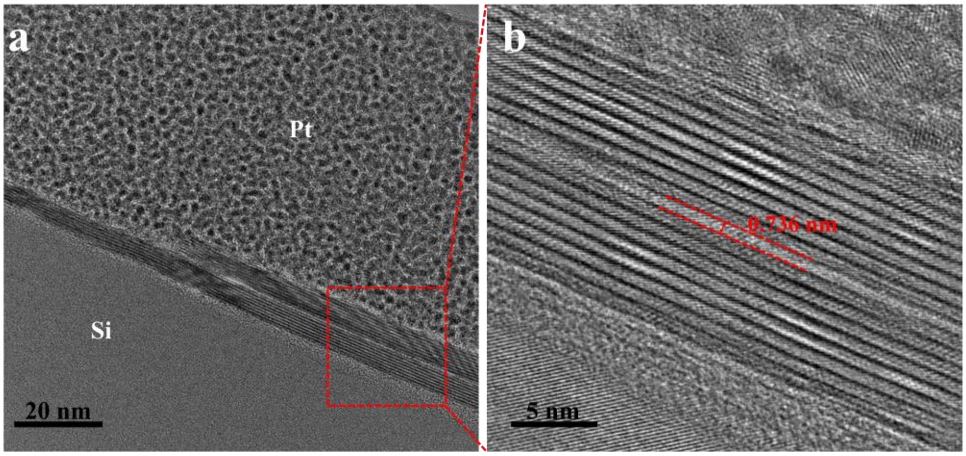

Supplementary Figure 9: The side-view HRTEM image of BiOCl MSL nanosheet. The cross section of the MSL was obtained by using the focused ion beam, in which procedure the measured micro area is protected by deposited platinum before being etched by gallium ions.

Supplementary Note 7

We have constructed the four-layer models in which two bilayer-nanosheets are stacked with twist angles. We have also constructed the 14-layer models of two 7-layer nanosheets as exactly synthesized experimentally. The results are shown the Supplementary Figure 10. Both models produce clear Moiré pattern.

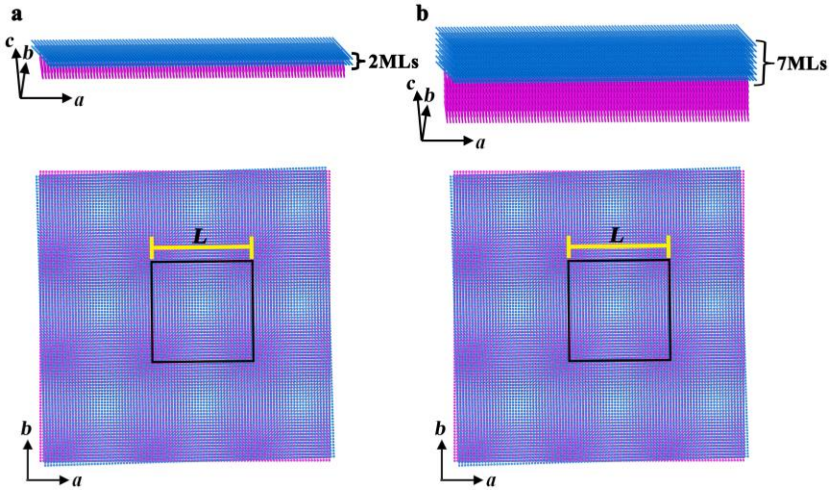

Supplementary Figure 10: 3D scheme and 2D perpendicular view of (a) the four-layer models in which two bilayer-nanosheets are stacked with twist angles, and (b) the 14-layer models in which two 7-layer-nanosheets are stacked with twist angles. Both models produce clear Moiré pattern.

Supplementary Note 8

Based on a series of TEM images of BiOCl nanosheets at different reaction times (Figure 2a), the growth mechanism of the spiral BiOCl nanosheets is proposed in Supplementary Figure 11. As in usual solvothermal reactions, isolated irregular nanosheets are formed at the preliminary stage (step I). During the assembly of isolated nanosheets, the line of screw dislocation forms. Under low supersaturation condition, which is the key to grow spirals [see Morin et al., Science 328, 476–480 (2010) and Meng et al., Accounts of Chemical Research 46, 1616-1626 (2013)][1, 2], the screw dislocation line creates step edge upon intersection with a crystal surface (step II). Then, more reaction atoms are added on the step edge, and a new surface layer is created without need to overcome the energy barrier (step III). Finally, the spiral Hanoi tower-like structures are formed along the screw-dislocation axis (step IV and V). This growth mechanism is described in the known the Burton-Cabrera-Frank model[1, 3]. In the process, the low supersaturation is essential for the screw dislocation to promote spiral crystal growth. Under the high supersaturation condition, the layer-by-layer dendritic growth mode is activated, which usually yields crystals with rough surfaces and fractal patterns.

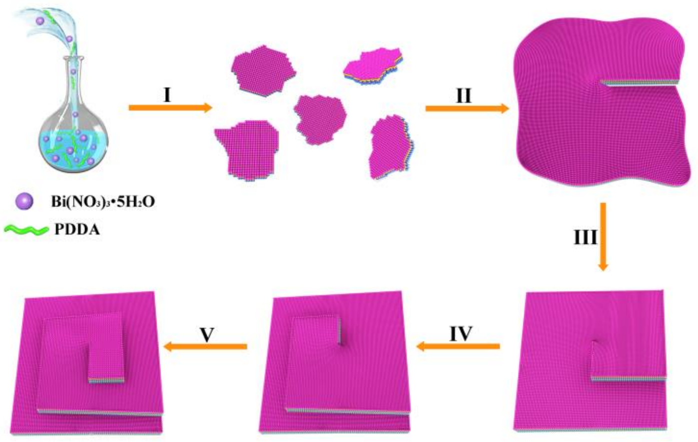

Supplementary Figure 11: Schematic modes showing the growth process of the spiral BiOCl nanosheets.

Supplementary Note 9

According to the classical crystal growth theory, the low supersaturation is essential for the screw dislocation to promote spiral crystal growth. Under the high supersaturation condition, the layer-by-layer, dendritic growth mode is activated, which usually yields crystals with rough surfaces and fractal patterns. To get the benchmark of low supersaturation condition in the spiral BiOCl nanosheets, we performed more synthesis trials by changing the concentration of the precursors ($Bi(NO_3)_3$, PDDA), as shown in the Supplementary Figure 12. When the concentration of $Bi(NO_3)_3$ is higher than 0.4 mM and the concentration of PDDA higher than 2.25 wt%., the spiral nanosheets of BiOCl cannot be obtained. A further increase in the concentrations of $Bi(NO_3)_3$ to be 1.6 mM and the concentration of PDDA to be 9 wt%, dendritic phase is formed.

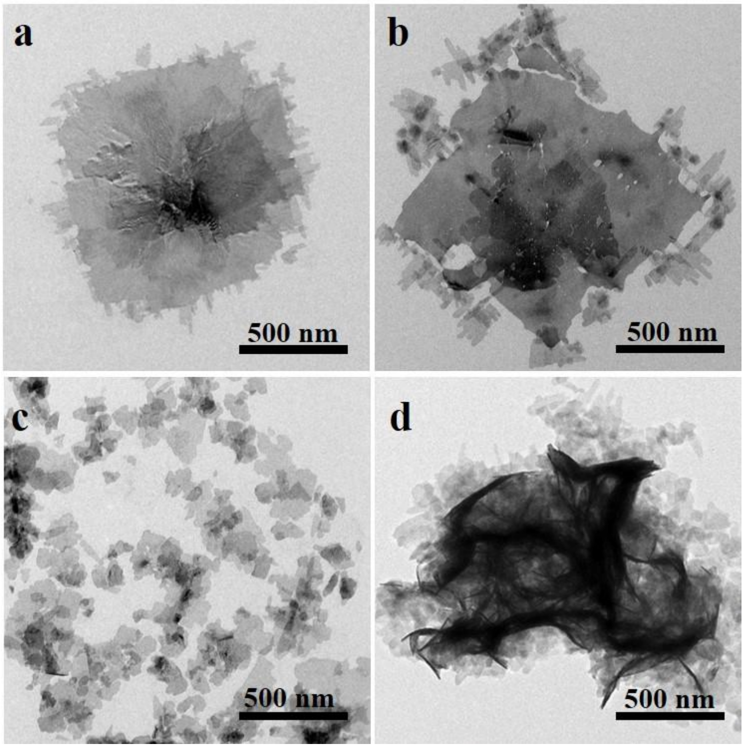

Supplementary Figure 12: a-d) Typical TEM images of the products obtained with the precursors concentrations of) $Bi(NO_3)_3$ (0.4 mM), PDDA (2.25 wt%) b) $Bi(NO_3)_3$ (0.8 mM), PDDA (4.5 wt%) c) $Bi(NO_3)_3$ (1.2 mM), PDDA (6.75 wt%) and d) $Bi(NO_3)_3$ (1.6 mM), PDDA (9 wt%).

Supplementary Note 10

UV/vis absorption spectra of the spiral BiOCl nanosheets are measured at different reaction times. The samples from three batches of growth processes are selected for the measurements (Supplementary Figure 13a, 13c, 13e). The standard Tauc plots are used to obtain band gap values (Supplementary Figure 13b, 13d, 13f). Since BiOCl is known to have an indirect band gap, the relationship of $\alpha(E_{\mathrm{ph}})^{1/2} \propto E_{ph} - E_g$ (where α, E_{ph}, and E_g represent absorption coefficient, photon energy, band gap) is adopted. The results are summarized in Figure 2c of the main text.

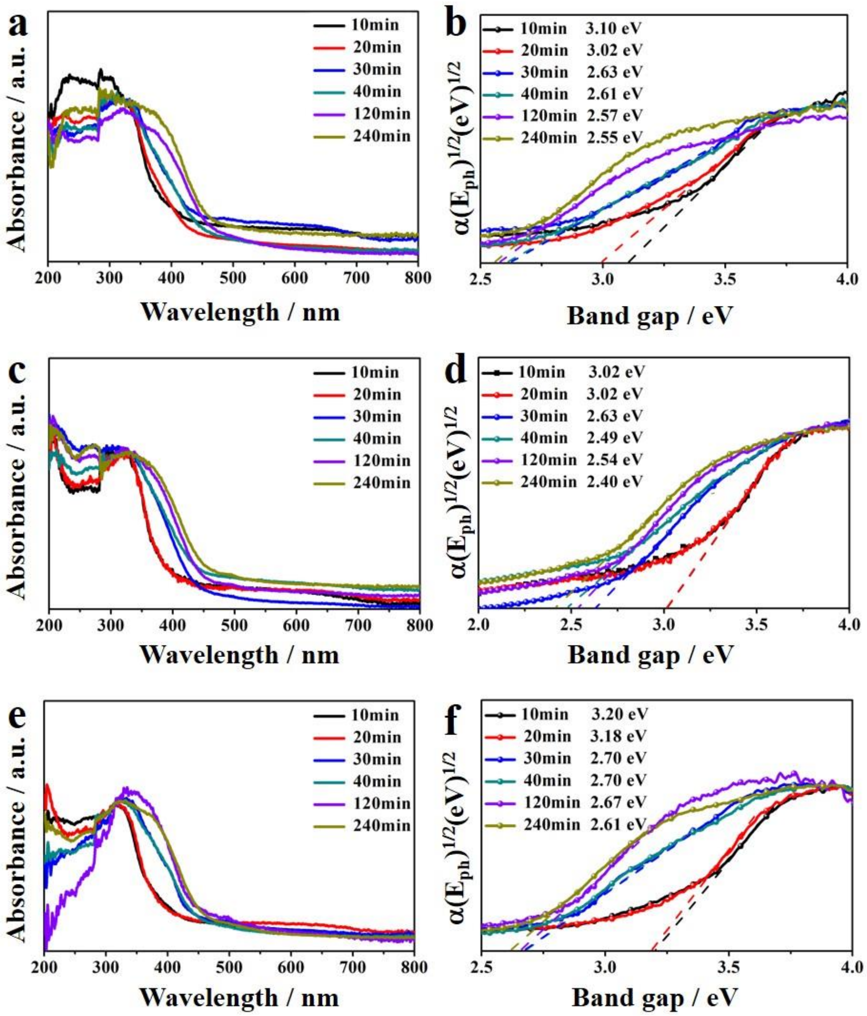

Supplementary Figure 13: Left panels: UV/vis absorption spectra of the spiral BiOCl nanosheets at different reaction times. (a,c,e) correspond to the samples from three batches of growth processes. Right panels: (b,d,f) Tauc plots of absorption spectra data to obtain band gap values.

Supplementary Note 11

We measure the ultraviolet photoelectron spectra (UPS) of the spiral BiOCl nanosheets to characterize the change of the valence band maximum (VBM) upon appearance of MSLs. The measurements are carried out at the reaction time of 10 min and 30 min, as shown in Supplementary Figure 14. The VBM energy (E_{VBM}) can be calculated from the UPS data following the formula of $E_{VBM} = h\nu - (E_{cutoff} - E_{Fermi})$, where $h\nu$ is the incident photon energy (21.22 eV here), E_{cutoff} is the secondary electron cutoff energy (taken from Supplementary Figure 14a), and E_{Fermi} is the onset energy relative to the Fermi level (taken from Supplementary Figure 14b). The calculated VBM energies of the BiOCl nanosheets at 10 min and 30 min are 6.87 and 6.45 eV, respectively. Therefore the appearance of MSLs leads to a 0.42 eV up-shift of the VBM.

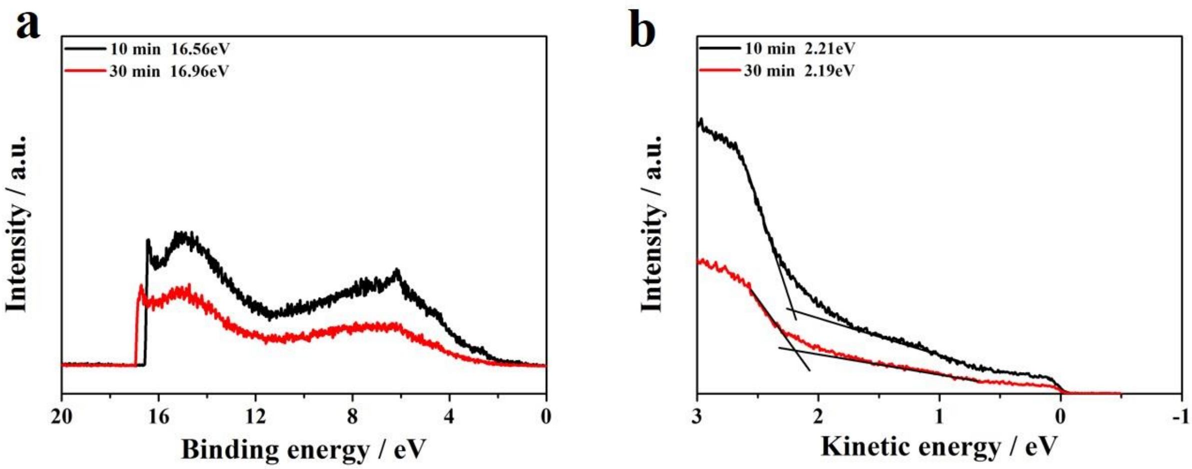

Supplementary Figure 14: a) Ultraviolet photoelectron spectra (UPS) in the secondary edge region of the spiral BiOCl nanosheets at the reaction time of 10 min and 30 min. b) Low energy onset region of the UPS of the nanosheets. From (a) one can obtain the secondary electron cutoff energy (E_{cutoff}) and from (b) one can obtain the onset energy relative to the Fermi level (E_{Fermi}). These data are then used to calculate the valance band maximum (VBM).

Supplementary Note 12

The electron paramagnetic resonance (EPR) technique is known as a sensitive spectroscopic tool to monitor defect formation process in materials. To detect the possible existence of oxygen vacancies in BiOCl nanosheets, we perform the low-temperature EPR technique as in the Supplementary Figure 15. No observable signals that are related to oxygen vacancies and other defects are observed during our preparation of BiOCl nanosheets.

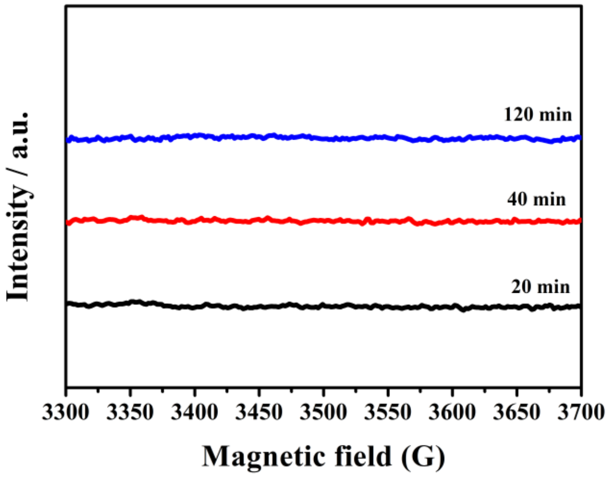

Supplementary Figure 15: Low-temperature EPR spectrum of spiral BiOCl nanosheets at different reaction times.

Supplementary Note 13

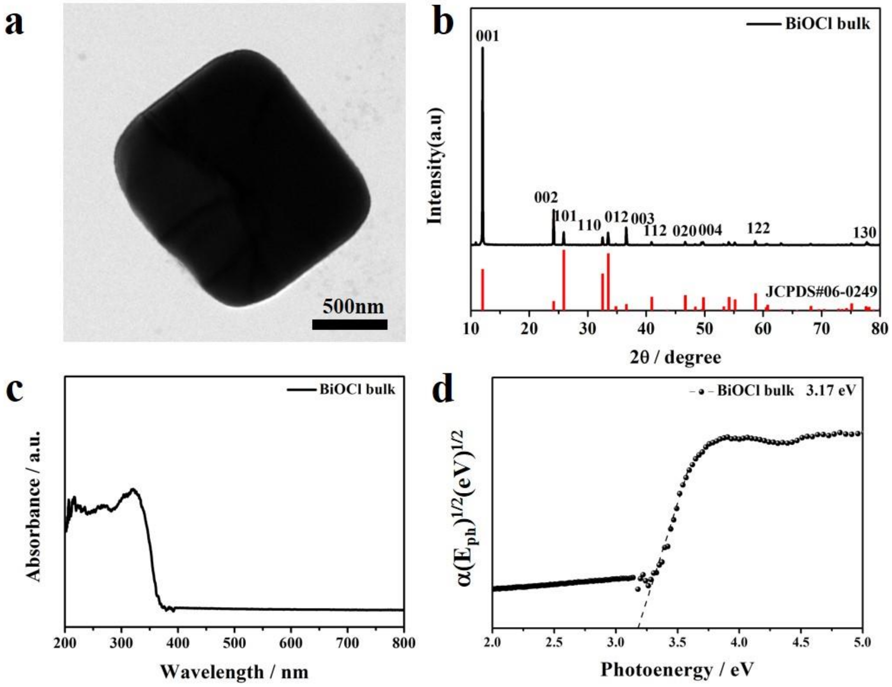

Supplementary Figure 16: Characterizing properties of BiOCl bulk sample: a) TEM image, b) XRD pattern, compared with the benchmark data of BiOCl from the JCPDS card (in red), c) UV/vis absorption spectrum, and d) Tauc plot of absorption spectrum to obtain band gap value. The relationship for indirect band-gap of $\alpha(E_{ph})^{1/2} \propto E_{ph} - E_g$ (where α, E_{ph}, and E_g represent absorption coefficient, photon energy, band gap) is adopted.

Supplementary Note 14

Four bilayer MSL model with the twist angles of 1.6°, 2.0°, 2.5°, and 3.0° are also shown in the Supplementary Figure 17. The period of squared Moiré patterns corresponds to twist angle of the bilayer MSL model.

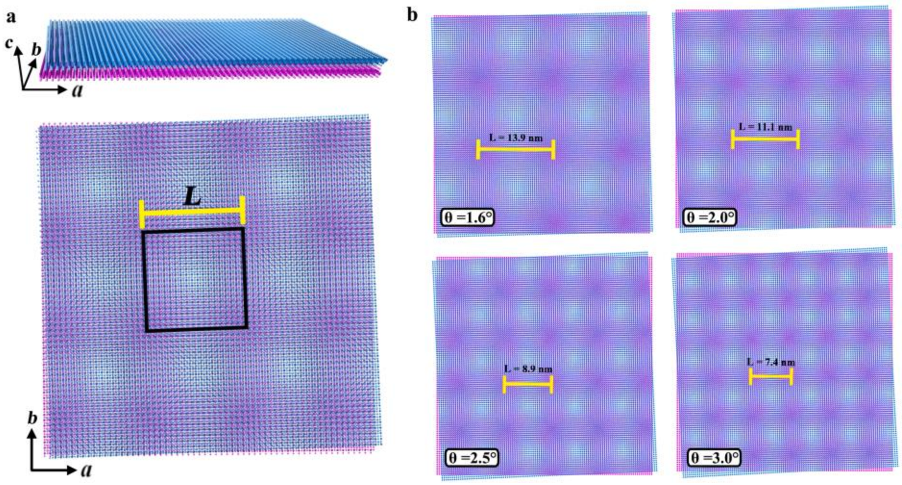

Supplementary Figure 17: a) 3D scheme and 2D perpendicular view of the bilayer Moiré superlattice model used in first-principles calculations. b) 2D perpendicular view of the bilayer Moiré superlattices with twist angle of 1.6°, 2.0°, 2.5°, and 3.0°, respectively.

Supplementary Note 15

We performed the aberration-corrected HADDF-STEM analysis (Supplementary Figure 18) to intuitively observe the atomic structure of the difference regions of BiOCl MSL. From the high-resolution enlarged HAADF-STEM image in Supplementary Figure 18e-f, we can see in detail the explicit atomic arrangements of the largest Bi atoms, which also agrees with the atomic structures in HH-stack and AH-stack region from the simulation results (Supplementary Figure 18g-h).

We employed the aberration-corrected HADDF-STEM measurement focusing on the nanosheet edge regions. The results are shown in the Supplementary Figure 19. This allows us to clearly resolve atomic-scale structures on the step sites to detect the possible existence of high-indexed facets. The high-indexed facets are clearly seen and marked near the nanosheet edges where exhibit high density of low-coordinate (003) atomic steps and few cases of (101) atomic steps.

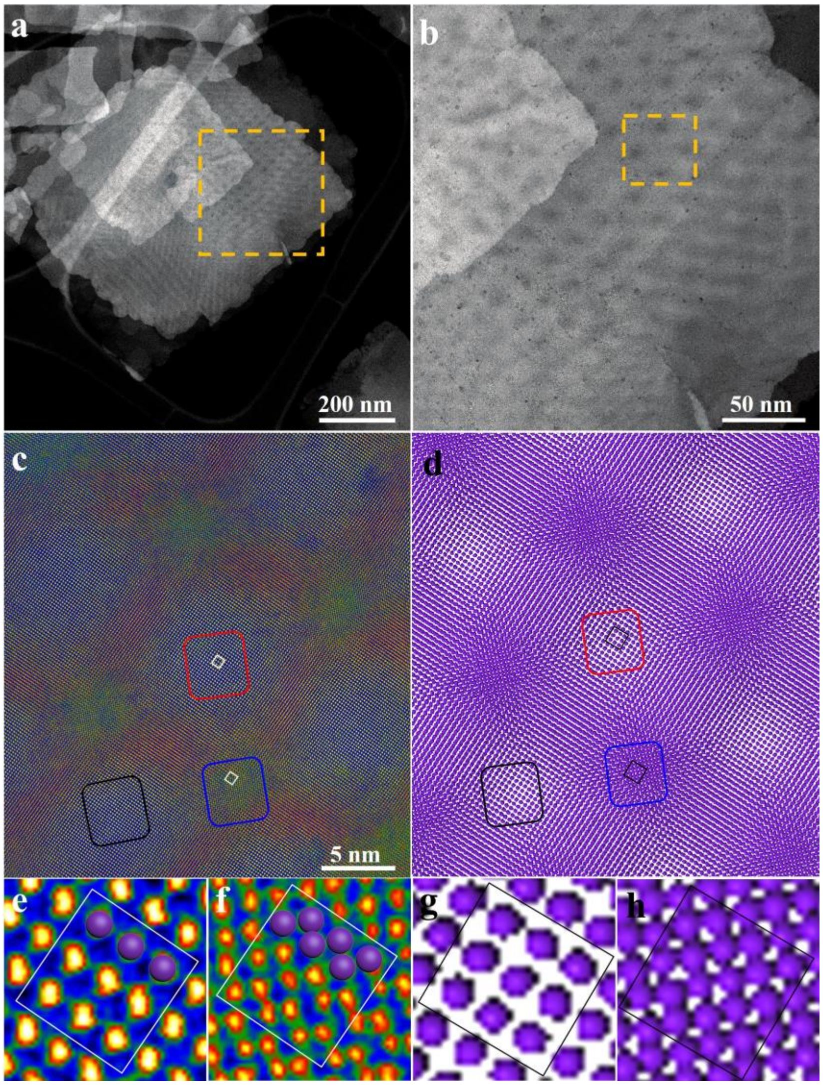

Supplementary Figure 18: a) Aberration-corrected HADDF-STEM image of BiOCl MSL. b) Amplified aberration-corrected HADDF-STEM image of selected region (in yellow) of (a) to clearly show profile of moiré pattern. c) The high-magnification contrast-false color HAADF-STEM image of selected region (in yellow) of (b) to clearly observe three distinct regions (named HH-stack, AH-stack, AA-stack structures in Fig. 3a). d) Perpendicular view of our simulated BiOCl bilayer model showing the MSL structures with twist angle. For clarity only Bi atoms are shown. e, g) Amplified contrast-false color HAADF-STEM image and simulated BiOCl bilayer model image in the square area of red square of (c), (d), respectively. f, h) Amplified contrast-false color HAADF-STEM image and simulated BiOCl bilayer model image in the square area of blue square of (c), (d), respectively.

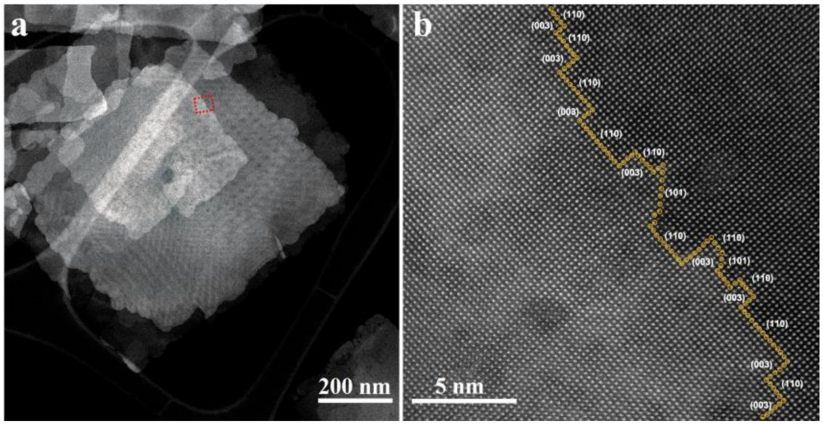

Supplementary Figure 19: a) Aberration-corrected HADDF-STEM image of BiOCl MSL. b) Amplified aberration-corrected HADDF-STEM image of selected region (in red) of (a) to clearly show the crystal faces on the step sites.

Supplementary Note 16

The densities of states and charge density differences of the bilayers BiOCl with HH-stack, AH-stack, AA-stack are now shown in the Supplementary Figure 20. We note that the non-periodic incommensurate stacking of BiOCl bilayers in our MSL model is approximated by using periodic boundary condition. The obtained results largely represent the local behavior in different regions of the MSL.

We calculate variation of band gaps throughout the constructed MSL (E_g(r)) in Figure 3a of the main text. This is done by taking the local atomic structure (*i.e.*, particular stacking pattern) at the position r. As shown in Supplementary Figure 21a, we calculate the particular stacking patterns in the irreducible triangle region (indicated in yellow) to extrapolate to the whole MSL. Interlayer distance of 0.736 nm, as obtained from the HRTEM and XRD measurements, is used. The band structures of three predominated bilayer structures, *i.e.*, the endpoints in the triangle of Supplementary Figure 21a, are shown in Supplementary Figure 21b.

From the inset of Figure 3d of the main text, we can see while the HH-stack structure shows almost unchanged band gap with varying interlayer distance *d*, the AA-stack structure demonstrates dramatic change in band gap with *d*. The underlying mechanism ascribes to the strong coupling among interlayer electronic states (*i.e.*, the antibonding interactions between the monolayer π(Cl-p_z) states) for the AA-stack structure. As illustrated in the main text, the band gap change can be ascribed to the shift of the valence band maximum (VBM) state. Supplementary Figure 22 shows the VBM partial charge density plots. For the HH-stack structure the VBM originates from the weak anti-bonding interaction between two monolayer anti-bonding σ*(Bi-*s*/Cl-$p_{x/y}$) states, $\Sigma^*(\sigma^*/\sigma^*)$, whereas for the AA-stack structure the VBM is made up of the strong anti-bonding interaction between two monolayer π(Cl-p_z) states, $\sigma^*(\pi/\pi)$.

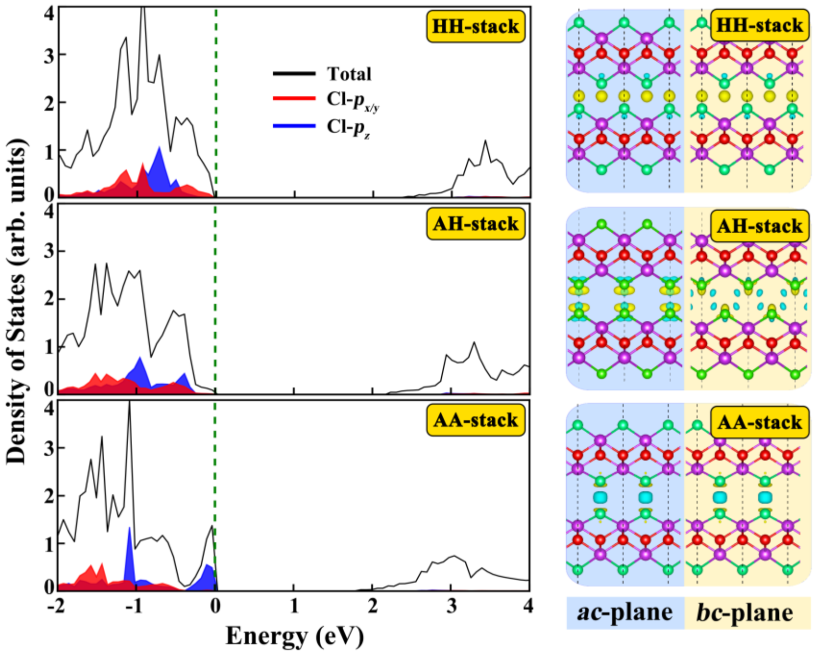

Supplementary Figure 20: The electronic densities of states (left panels) and charge density differences (right panels) of the BiOCl bilayers with HH-stack, AH-stack, AA-stack patterns under periodic boundary condition. For the charge density differences the isosurface value is 0.002 electrons/Å^3, and the charge accumulation and depletion are denoted in yellow and blue, respectively.

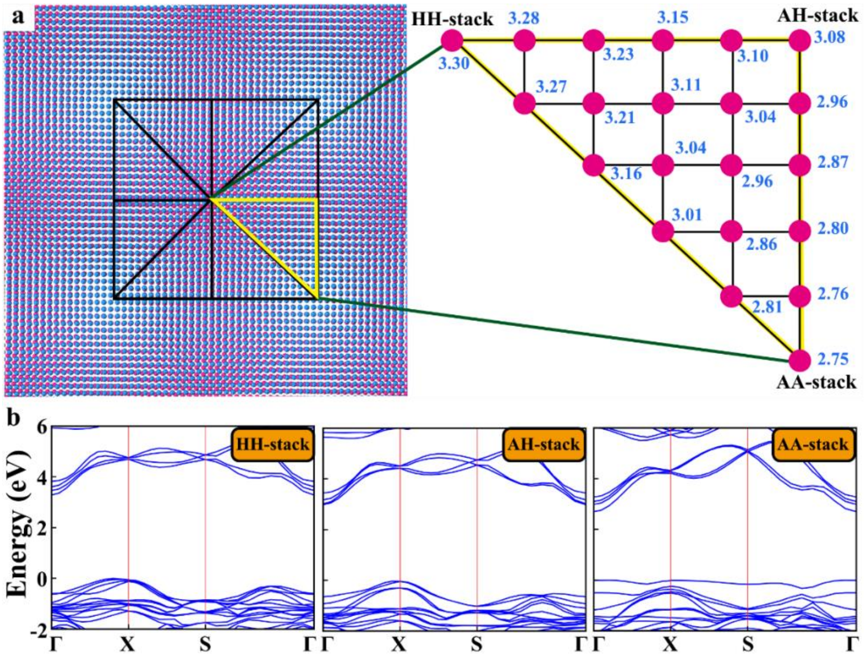

Supplementary Figure 21: a) Description of how the variation of band gaps throughout the MSL ($\mathbf{E_g(r)}$) in Figure 3d of the main text is calculated. Since the MSL has one fourfold rotation and two mirror planes symmetries, we can calculate the local atomic structures (*i.e.*, particular stacking patterns) in the irreducible triangle region (indicated in yellow) to extrapolate to the whole MSL. We adopt a 5x5 point meshes in the triangle, at which the calculated gap values are given

in the plot. b) Electronic band structures of three predominated bilayer structures with different stacking patterns (named HH-stack, AH-stack, AA-stack structures, see Figure 3c of the main text). The valence band maximum is set to energy zero.

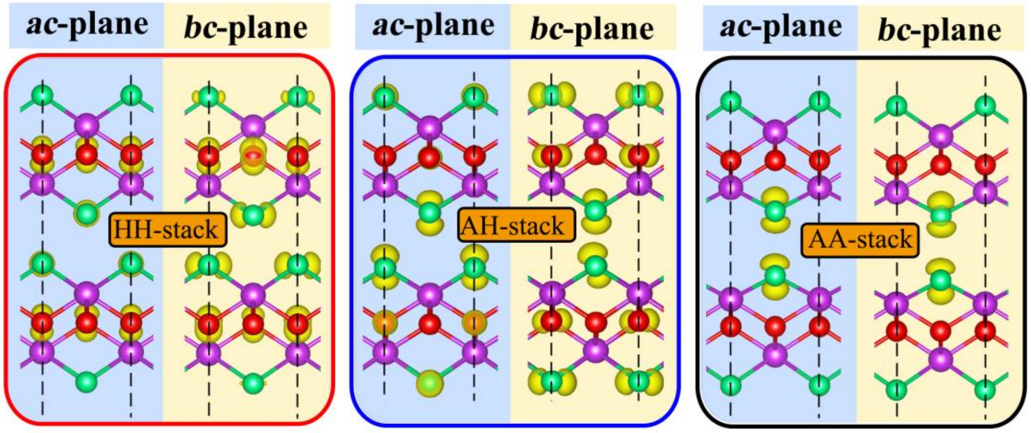

Supplementary Figure 22: The partial charge density (wavefunction square) of the valence band maximum states for the three structures with different stacking patterns. The isosurface value is set to 1×10^{-4} electrons/Å^3.

Supplementary Note 17

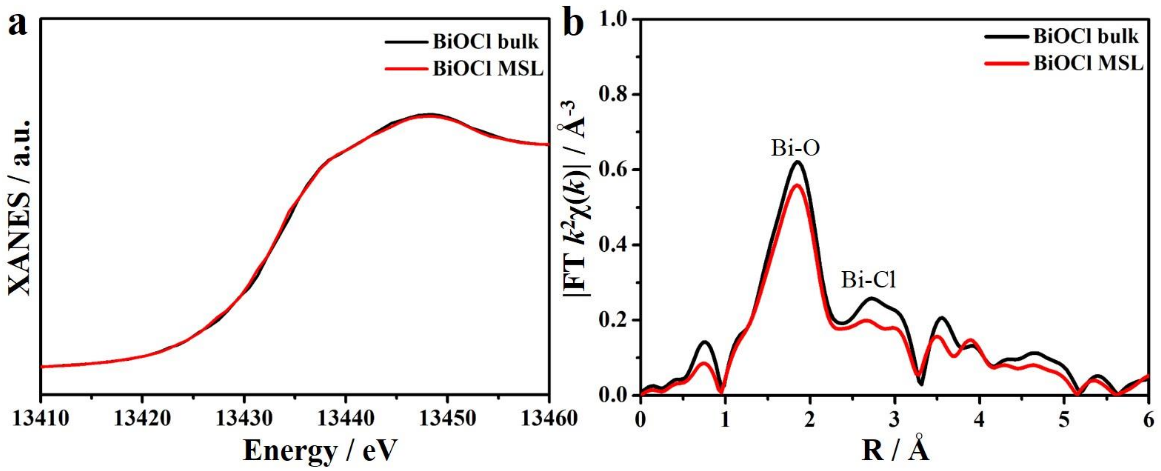

Supplementary Figure 23: a) Bi L_{III}-edge XANES spectra of BiOCl bulk and BiOCl MSL. b) Bi L_{III} -edge EXAFS spectra of BiOCl bulk and BiOCl MSL.

Supplementary Note 18

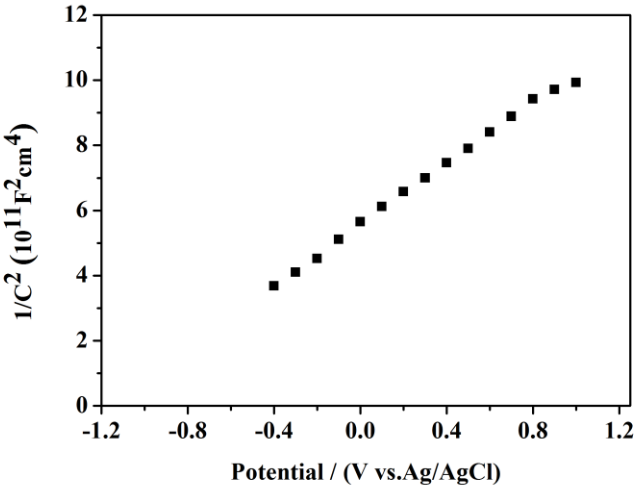

Supplementary Figure 24: Mott-Schottky analysis plots for BiOCl spiral nanosheet in a 0.5 M Na_2SO_4 aqueous solution. The positive slope of the 1^C^2–E plot suggests electrons as majority carriers (*i.e.*, n-type semiconductor behavior).

Supplementary Note 19

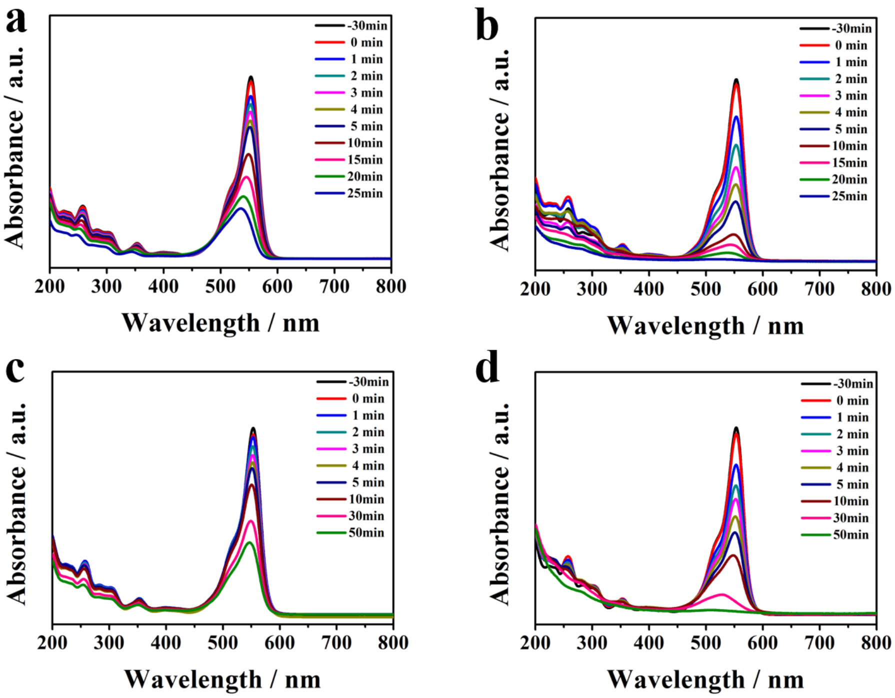

Supplementary Figure 25: Photocatalytic degradation of Rhodamine B with (a, c) BiOCl bulk and (b, d) BiOCl MSL nanosheets as catalysts. The Rhodamine B degradation is detected by the change in intensity of its characteristic peak of temporal UV-vis absorption spectrum. Measurements are taken under simulated solar irradiation (a, b) and visible light irradiation (c, d), respectively. Negative time denotes dark condition before light irradiation.

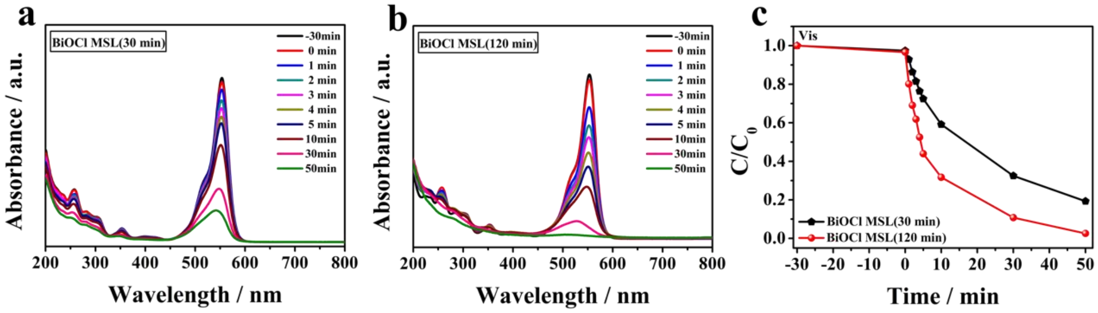

Supplementary Figure 26: (a,b) Photocatalytic degradation of Rhodamine B with BiOCl MSL (30 min) and BiOCl MSL (120 min) nanosheets as catalysts. The Rhodamine B degradation is detected by the change in intensity of its characteristic peak of temporal UV-vis absorption spectrum. Measurements are taken under visible light irradiation. Negative time denotes dark condition before light irradiation. c) Comparison of photocatalytic degradation rate of Rhodamine B versus time for the two samples.

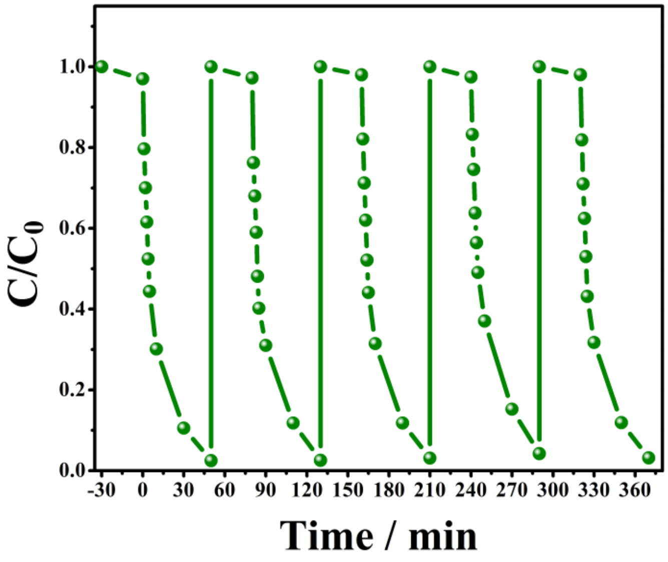

Supplementary Figure 27: The variation of Rhodamine B concentration (*C*/C_0) versus irradiation time with BiOCl MSL as catalysis in 5 successive cycles under visible light irradiation. The C_0 and *C* are the initial concentration (10 mg^L^{-1}) and the instant degradation concentration of Rhodamine B during the degradation process, respectively.

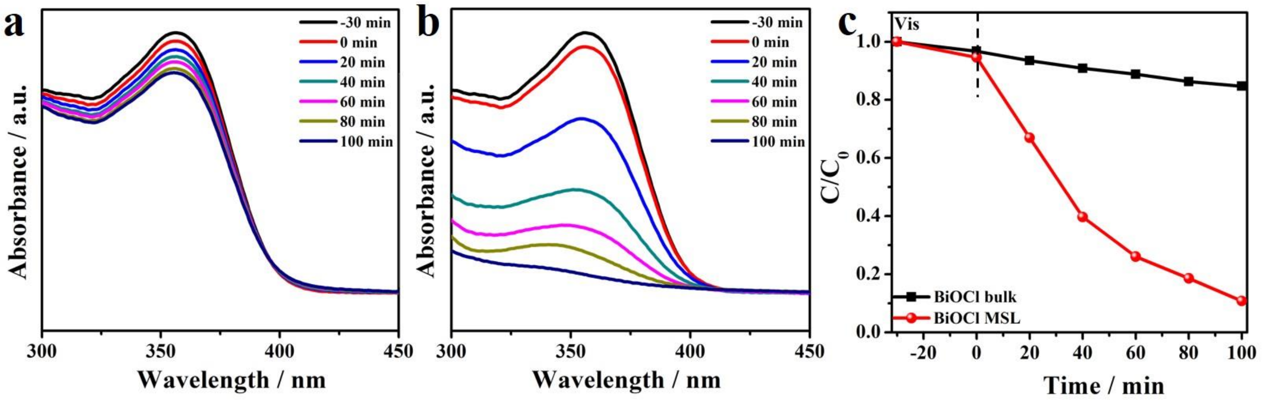

Supplementary Figure 28: Photocatalytic degradation of tetracycline with a) BiOCl bulk and b) BiOCl MSL nanosheets as catalysts. The tetracycline degradation is detected by the change in intensity of its characteristic peak of temporal UV-vis absorption spectrum. Measurements are taken under visible light irradiation. Negative time denotes dark condition before light irradiation. c) Photocatalytic degradation rate of tetracycline versus time with BiOCl bulk and BiOCl MSL nanosheets as catalysts.

Supplementary Note 20

We measure time-resolved photoluminescence (TRPL) spectra to investigate carrier dynamics of BiOCl MSLs. Measurement of BiOCl bulk sample is carried out for comparison. The emission decay data of BiOCl can be fitted by a biexponential function of $I(t) = B_1 e^{-t}/\tau_1 + B_2 e^{-t}/\tau_2$, where $I(t)$ is the decay of fluorescence intensity at time t, B_1 and B_2 are the preexponential factors, and the fast-process τ_1 relates to nonradiative decay, whereas the slow-process τ_2 represent radiative recombination of photogenerated carriers. The average carrier lifetime τ_{avg} is further evaluated by $\tau_{avg} = A_1\tau_1 + A_2\tau_2$ with component proportion magnitudes $A_1 = B_1\tau_1/(B_1\tau_1 + B_2\tau_2)$ and $A_2 = B_2\tau_2/(B_1\tau_1 + B_2\tau_2)$. The fitting results of BiOCl bulk, BiOCl MSL nanosheets measured at the reaction time of 30 min and 120 min are summarized in Supplementary Table 1.

Supplementary Table 1. The fitting results of the photoluminescence emission decay data by using the biexponential function. χ^2 depicts the fitting quality (χ^2 = 1 represents perfect fitting).

	B_1	τ_1 (ns)	A_1(%)	B_2	τ_2 (ns)	A_2(%)	τ_{avg} (ns)	χ^2
BiOCl bulk	2.25×10^5	2.82	99.5	284.13	11.21	0.5	2.86	0.998
BiOCl MSL (30 min)	6.79×10^4	3.78	98.3	336.44	13.19	1.7	3.94	0.997
BiOCl MSL (120 min)	3.04×10^4	5.27	95.8	254.16	27.64	4.2	6.21	0.998

Supplementary References

1. Morin, SA. et al. Mechanism and kinetics of spontaneous nanotube growth driven by screw dislocations. Science **328**, 476-480 (2010).

2. Meng, F. et al. Screw dislocation driven growth of nanomaterials. Acc Chem Res **46**, 1616-1626 (2013).

3. Burton, WK. et al. Role of dislocations in crystal growth. Nature **163**, 398-399 (1949).
