# Supplementary material for: Bottom-up growth of homogeneous Moiré superlattices in bismuth oxychloride spiral nanosheets
Source: Nat Commun. 2019 Oct 2;10:4472. doi: 10.1038/s41467-019-12347-7 (PMC6775108; doi:10.1038/s41467-019-12347-7)
Supplement: Supplementary file 2 — Peer Review [file 41467_2019_12347_MOESM2_ESM.pdf]

Reviewers' comments:

Reviewer #1 (Remarks to the Author):

This manuscript reports a facile bottom-up synthesis of homogeneous Moiré superlattices (MSL) based on BiOCl, which constitutes spiral-type nanosheets with 1.6~3.0° twist angles among adjacent sheets. The MSL exhibited a considerable band gap reduction of about 0.60 eV compared with bulk BiOCl, rendering BiOCl an optically active material in visible-light region. Furthermore, the carrier lifetime of MSL was also increased for more than two times. As a result, the MSL showed much enhanced photocatalytic activity by comparison with bulk BiOCl. The work is interesting although with few ambiguous claims. It is recommended for publication in Nature Communication for major revision. Specific comments are as follows.

1. How the bilayer MSL model for DFT calculation is constructed and whether the correctness of such constructing method is examined? 3D schemes indicating the stacking of constituting two layers and atomic structure of the unit cell of BiOCl Moiré pattern should be provided. In addition, the as-claimed squared Moiré patterns with period L from 7.7 nm to 14.3 nm should be constructed using the bilayer MSL model with corresponding twist angle ranging from 3.0° to 1.6° (Fig. 1 and S3–S4).
2. Could only bilayer model cause the Moiré pattern? and how about four-layer model of two bilayer nanosheets or six-layer model of two three-layer nanosheets with twist angles since the AFM image of MSL indicating constituting BiOCl nanosheets are not monolayers in thickness (Fig. S1)?
3. If the MSL was not comprised of bilayer BiOCl nanosheets, the distance of 0.736 nm in Fig. 1i would not be the as-claimed distance of AA-stack but the length of c axis for BiOCl lattice. Furthermore, the influence of AA-stack to bandgap reduction would be reduced, then how to understand the huge bandgap reduction of ~0.6 eV for BiOCl, while that for graphene MSL was merely 0.05 eV?
4. EG reduction would cause oxygen vacancies with concentrations increased by reduction time during the BiOCl preparation, and the oxygen vacancies of BiOCl would also lead to bandgap reduction and increase in lifetime of charge carriers. How to distinguish the role of oxygen vacancies from that of MSL?
5. The region of HH-stack, AH-stack, AA-stack bilayers BiOCl are isolated with each other. Under the excitation of visible light, how are the low energy carriers in AA-stack transfer to the surface of BiOCl through HH-stack region?
6. In the case of periodic boundary condition, if it is suitable to calculate the band gap, what are density states and charge density differences of non-periodic bilayers BiOCl with HH-stack, AH-stack, AA-stack?
7. The bulk BiOCl has the ability to degrade RhB under visible light with dye sensitization of RhB. To avoid confuision, the authors should detect the visible-light photocatalytic activity of BiOCl MSLs in another way, for example, the degradation of colorless molecules.
8. In the experimental part, the BiOCl MSL was bottom-up chemical growth of spiral-type nanosheets with 1.6∼3.0° twist angles among adjacent sheets, however, in the modeling section, the BiOCl spiral bilayer structure with the 1.7° twist angle was constructed, one was the adjacent sheet (each sheet consists of seven BiOCl atomic monolayers), and the other was the bilayer structure. This description made the modeling and the experiment seriously inconsistent. If this modeling is reasonable, the authors are recommended to change the description of bilayers to represent the bottom layer of the upper sheet and the top layer of the lower sheet for better understanding.

Reviewer #2 (Remarks to the Author):

The authors report on the synthesis of bismuth oxychloride spiral nanosheets with small interlayer twist angles using a facile chemical approach. The resultant Moiré superlattice structure lead to changes in their properties as compared to bulk HH-stacked structure such as reduced bandgap, enhanced carrier lifetime and photocatalytic activity. Overall this work has interesting results which broaden the understanding of screw-dislocation driven growth of 2D nanomaterials and probed into

their unique properties. Based on novelty and the results gathered, the reviewer will consider publication after the authors consider these questions:

1. There is a lack of introduction to BiOCl and other 2D materials having screw-dislocation driven growth. What is the current research status of BiOCl nanosheets? (i.e., synthesis methods, properties and applications) This would make the manuscript much more comprehensive to the wider field.

2.Are all the synthesized BiOCl spiral nanosheets formed with a small degree of interlayer twist angle? Why is it only unique to BiOCl and not observed for pervious 2D materials or nanoplates with spiral structures (or this should be present for other 2D materials as well)? Since the formation of interlayer twist is important here, its mechanism should be explained. Could this be associated to the strain generated by the screw-dislocation similar to "Eshelby twist" for 1D nanowires?

3.In Figure 2b, there are some peaks missing in their XRD spectra for 40 min and above. What does the missing peaks indicate?

4.The term "Real-time" in the heading "Real-time characterization of growth…" is misleading. TEM was done after synthesis on transferred samples, not in "real-time".

5.For multiple spiraling layers besides two layers, is there a constant interlayer twist between the successive layers? For example, does the periodicity of the Moiré pattern decreases with increasing layers? What effect does this have towards its emergent properties?

Reviewer #3 (Remarks to the Author):

In this work, 2D Moiré superlattices (MSLs) BiOCl was synthesized via solvothermal reaction, which was considered to follow a screw-dislocation driven mechanism. The fabrication of BiOCl MSLs leads to strong modulation of optoelectronic properties with 0.6 eV band gap reduction and substantial carrier lifetime increase, which may cause the enhanced visible-light photocatalytic activity. First-principles calculations further revealed that the locally enhanced inter-layer coupling effect associated with the Moiré potential modulation may play an important role in the modulation of optoelectronic properties. Only after addressing the following questions can this work be published.

1. I am wondering if surfaces of BiOCl are clean enough, without capping agents residuals. If PDDA caps on the surfaces of BiOCl, how to remove it? Whether PDDA participate in catalytic process?

2. There are many methods to modify BiOCl, such as doping and heterostructures regulation. Compared to the traditional methods, what is the unique advantage of screw dislocation?

3. For the growth mechanism, more quantified information is need. For example, the authors claim the low supersaturation condition responsible for the screw dislocation, then how low, or what is the benchmark? Why low supersaturation condition is important?

4. TEM images in the manuscript just exhibited one single BiOCl nanosheet. TEM images that show more nanosheets are needed to further prove the monodispersity. Moreover, some cracks and irregular edges appeared in TEM images. What caused the existence of this kind of structure?

5. Cs-corrected HADDF STEM was widely used to characterize nanoscale structure down to atomic level. In this work, Cs-corrected HADDF-STEM should be used to characterize the differences among HH-stack, AH-stack, and AA-stack. What was the atomic arrangement in the uniaxial-oriented of BiOCl MSLs? Would strain exist in the uniaxial-oriented of BiOCl MSLs?

6. I am wondering if high-indexed facets could exist on step sites in the spiral nanosheets. If so, please index the specific facets.

7. The authors mentioned bidirectional growth with AFM images. Are the growth speeds in two directions the same?

8. The manuscript discussed HH-stack and AA-HH-stack a lot, but did not pay too much attention on the HA-stack structure. Could the authors explain the unique properties of HA-stack and how it worked in this system?

9. Whether the L-edge of XANES and EXAFS for Bi in spiral nanosheets and bulk BiOCl are the same?

10. What are the reaction sites on BiOCl MSLs during catalytic degradation, the oriented axis, high-indexed facets, edge sites, or others?

Point-by-point Responses to Reviewers' Comments

Note: *The Reviewers' Comments are with italic,* our Responses are in blue, the new changes made in the manuscript are in red.

<u>Reviewer #1</u>

(1) Referee: *This manuscript reports a facile bottom-up synthesis of homogeneous Moiré superlattices (MSL) based on BiOCl, which constitutes spiral-type nanosheets with 1.6~3.0° twist angles among adjacent sheets. The MSL exhibited a considerable band gap reduction of about 0.60 eV compared with bulk BiOCl, rendering BiOCl an optically active material in visible-light region. Furthermore, the carrier lifetime of MSL was also increased for more than two times. As a result, the MSL showed much enhanced photocatalytic activity by comparison with bulk BiOCl. The work is interesting although with few ambiguous claims. It is recommended for publication in Nature Communication for major revision. Specific comments are as follows.*

Reply: We thank the referee for the positive comments. In this revised version, we have made extensive revisions, added further experimental measurements and theoretical calculations to address the referee's questions below and strengthen the major points of this work.

(2) Referee: *How the bilayer MSL model for DFT calculation is constructed and whether the correctness of such constructing method is examined? 3D schemes indicating the stacking of constituting two layers and atomic structure of the unit cell of BiOCl Moiré pattern should be provided. In addition, the as-claimed squared Moiré patterns with period L from 7.7 nm to 14.3 nm should be constructed using the bilayer MSL model with corresponding twist angle ranging from 3.0° to 1.6° (Fig. 1 and S3–S4).*

Reply: In response to the referee's comments, we have now provided our constructed 3D scheme for the bilayer Moiré superlattice (MSL) model in DFT calculations as the new Fig. 1k. We have also revised Fig. 1j to clearly indicate the actual structure of our

experimentally synthesized BiOCl nanosheet. They are shown also as Fig. R1 below for the convenience of the referee. The specific atomic structures within the unit cell of the MSL are shown in Fig. 3a and 3c. The squared Moiré patterns with the period *L* from 7.7 nm to 14.3 nm and the corresponding twist angle from 3.0° to 1.6°, constructed using the bilayer MSL model, are now provided in the new Fig. S16 (shown also as Fig. R2 below). Four cases with the twist angles of 1.6°, 2.0°, 2.5°, and 3.0° are shown in this figure.

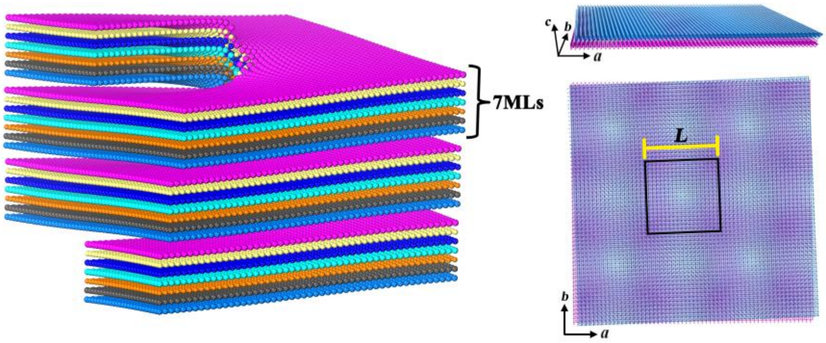

Figure R1: (Figures 1j and 1k) (j) Schematic of the grown BiOCl spiral nanosheets driven by the screw-dislocation. Each nanosheet consists of seven BiOCl monolayers (only O atoms are shown, in different colors). (k) 3D scheme and 2D perpendicular view of the bilayer Moiré superlattice model used in first-principles calculations. To clearly demonstrate Moiré pattern, only O atoms are shown and the upper and lower layers are shown in dodgerblue and purple, respectively. The period of Moiré superlattice *L* is indicated.

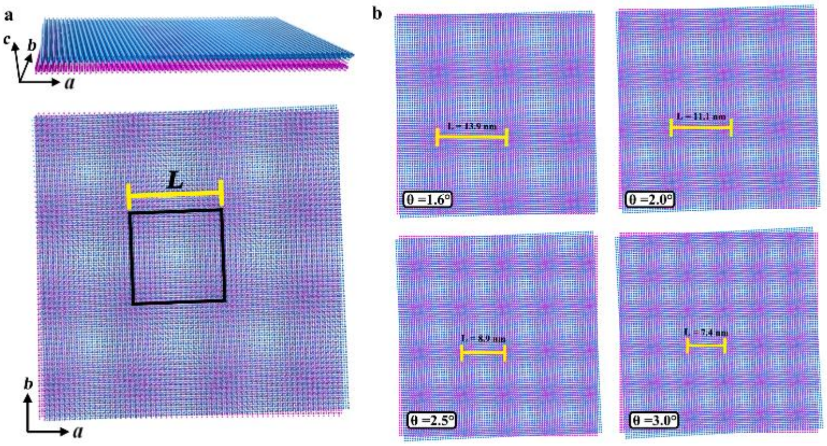

Figure R2: (Figure S16) a) 3D scheme and 2D perpendicular view of the bilayer Moiré superlattice model used in first-principles calculations. b) 2D perpendicular view of the bilayer

Moiré superlattices with twist angle of 1.6°, 2.0°, 2.5°, and 3.0°, respectively.

The reasonableness of our constructed model can be seen from the fact that the MSL and the resulted functional properties that we observe are due to the incommensurate structure formed between two outmost layers with thicker inert sections away from this. Experimentally, the twisting is observed between the adjacent nanosheets, each composed of seven BiOCl monolayers. The incommensurate structure occurs between the bottom monolayer of the upper nanosheet and the top monolayer of the lower nanosheet. The key of the MSL is spatial in-plane modulation of atomic-scale structures, which leads to a spatial modulation of electronic structure and related properties. Such spatial modulation is directly related to the two outmost BiOCl monolayers stacked with a twist angle. The inner BiOCl layers of the nanosheets do not (or barely) contribute to such spatial modulation. Hence, our constructed model, which is tailored for DFT calculations, captures the key structural features that are responsible for the spatial modulation of physical properties of interest. To make this point clearer, we have added the above discussion into p. 6, 2nd paragraph of the revised manuscript.

(3) Referee: *Could only bilayer model cause the Moiré pattern? and how about four-layer model of two bilayer nanosheets or six-layer model of two three-layer nanosheets with twist angles since the AFM image of MSL indicating constituting BiOCl nanosheets are not monolayers in thickness (Fig. S1)?*

Reply: This question is partially related to the previous comment about the MSL structure. To address it, we have now constructed the four-layer models in which two bilayer-nanosheets are stacked with twist angles. We have also constructed the 14-layer models of two 7-layer-nanosheets as exactly synthesized experimentally. The results are shown in the new Fig. S9 (shown also as Fig. R3 below for the convenience of the referee). Both models produce clear Moiré pattern. Accordingly, we now add the following clarification into p. 7 line 1 of the revised manuscript:

"In analogy to the usual MSLs formed by bilayers[3-9], such incommensurately

stacking between two nanosheets with multiple monolayers indeed leads to formation of MSLs (Supplementary Fig. 9)"

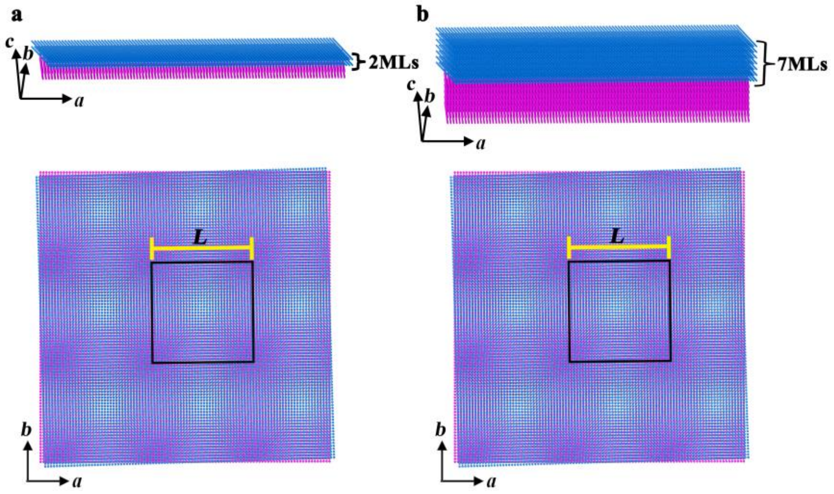

Figure R3: (Figure S9) 3D scheme and 2D perpendicular view of (a) the four-layer models in which two bilayer-nanosheets are stacked with twist angles, and (b) the 14-layer models in which two 7-layer-nanosheets are stacked with twist angles. Both models produce clear Moiré pattern.

(4) Referee: *If the MSL was not comprised of bilayer BiOCl nanosheets, the distance of 0.736 nm in Fig.1i would not be the as-claimed distance of AA-stack but the length of c axis for BiOCl lattice. Furthermore, the influence of AA-stack to bandgap reduction would be reduced, then how to understand the huge bandgap reduction of ~0.6 eV for BiOCl, while that for graphene MSL was merely 0.05 eV.*

Reply: Our synthesized BiOCl MSLs are formed by the twisted stacking of two 7-monolayers nanosheets. The distance (~0.736 nm) between the two adjacent nanosheets is determined from the AFM step height image (Fig. S1) and TEM measurement (Fig. 1i). This distance is equal to the length of *c* axis for BiOCl lattice as noticed by the referee. The inset is now added to Fig.S1b (shown also as Fig. R4 below for the convenience of the referee) to illustrate this point more explicitly.

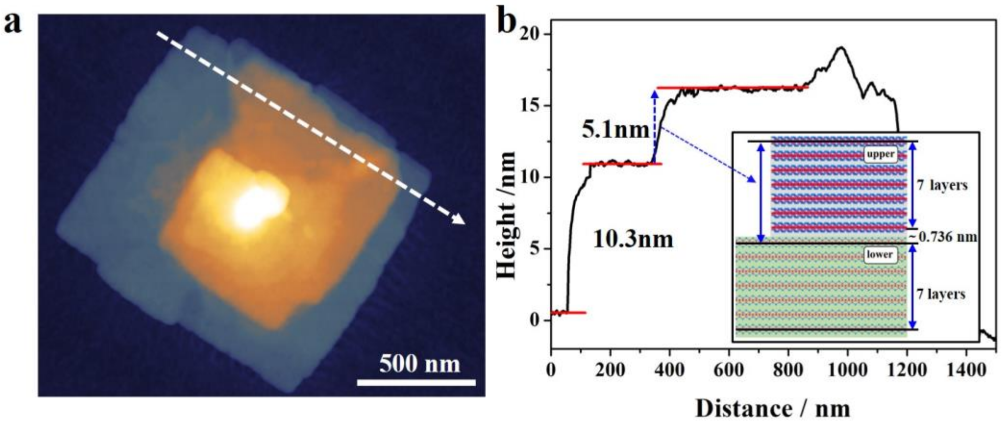

Figure R4: (Figure S1) a) AFM height-sensor micrograph of an individual BiOCl nanosheet. b) One-dimensional step height profile along the dash arrow line in (a). The inset indicates how the distance (~0.736 nm) between the two adjacent nanosheets is determined.

In our DFT calculations, the bilayer MSL model adopts the experimentally determined distance between the two adjacent nanosheets (~0.736 nm). The predicted 0.55 band gap reduction agrees with the experimentally observed value of ~0.6 eV. Such large band gap reduction is traced down to the AA-stack region of the MSL, which sustains the shorter interlayer distance than its equilibrium condition. Based on our calculation, the equilibrium interlayer distance for the AA-stack is as large as 0.877 nm. This originates from the AA-stack having crystal structure with the head-to-head arrangement of the Cl atoms from adjacent layers (Fig. 3c). Such shorter interlayer distance substantially enhances the interlayer coupling, upshifts the valence band edge, and reduces the band gap. This has been discussed in detail in the section of Origin of band-gap reduction in the manuscript.

(5) Referee: *EG reduction would cause oxygen vacancies with concentrations increased by reduction time during the BiOCl preparation, and the oxygen vacancies of BiOCl would also lead to bandgap reduction and increase in lifetime of charge carriers. How to distinguish the role of oxygen vacancies from that of MSL?*

Reply: To address the referee's comment, we have performed new experiments using

low-temperature electron paramagnetic resonance (EPR) technique to detect the possible existence of oxygen vacancies in BiOCl nanosheets. The EPR technique is known as a sensitive spectroscopic tool to monitor defect formation process in materials. The EPR spectra of the nanosheets at different reaction times are illustrated in the newly added Fig. S14 (shown also as Fig. R5 below for the convenience of the referee). The EPR spectra show no observable signals that are related to oxygen vacancies and other defects during our preparation of BiOCl nanosheets. This is consistent with the fact that EG was mainly used as a solvent not as a reductant during the synthesis of BiOCl samples [Please refer to Zhang, et al., J. Phys. Chem. C, 2008, 112, 747-753].

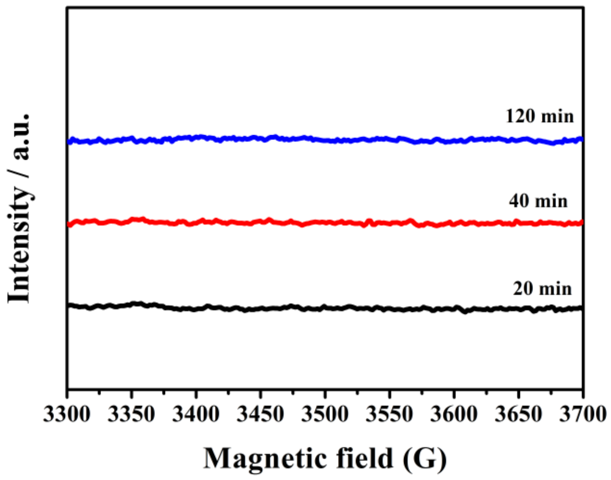

Figure R5: (Figure S14) Low-temperature EPR spectrum of BiOCl MSL.

Accordingly, in p. 8 line 24 of the revised manuscript we have now added the following sentence:

"The low-temperature EPR measurements of BiOCl nanosheets at different reaction times (Supplementary Fig. 14) show no observable signal, which excludes the role of easily formed oxygen vacancies as the origin of the change in optoelectronic properties."

(6) Referee: *The region of HH-stack, AH-stack, AA-stack bilayers BiOCl are isolated with each other. Under the excitation of visible light, how are the low energy carriers*

in AA-stack transfer to the surface of BiOCl through HH-stack region?

Reply: We thank the referee for the insightful comment. With emergence of the MSL, photo-induced electron and hole carriers are expected to show feasible transfer behaviors by consideration of the two aspects: (i) Based on the calculated conduction band minimum (CBM) change from the AA-stack to HH-stack bilayers (see Fig. 3g), we can see that the CBM decreases slightly from AA-stack to HH-stack. This means that electrons tend to flow from the AA-stack toward HH-stack regions in the MSL. (ii) The calculated valence band maximum (VBM) change from the AA-stack to HH-stack bilayers (Fig. 3g) indicates a hole localization tendency at the AA-stack region, as pointed out by the referee, which is also evidenced by the scattering-type scanning near-field optical microscopy (s-SNOM) results (Fig. 4d). However, we would like to draw the reviewer's attention to the fact that the regions of AA-stack, HH-stack, and intermediate AH-stack are actually not isolated in sample, but show gradually varying nature (see Fig. 3a). The corresponding VBMs also vary gradually from one region to the other (see also Fig. 3g). The gradually varying energy levels of hole states, in accompany with the existing concentration gradient among different regions, would result in reasonable hole transfer out of the AA-stack region. Pertinent discussion on the above has been added into p. 13 2nd graph of the revised manuscript.

(7) Referee:: *In the case of periodic boundary condition, if it is suitable to calculate the band gap, what are density states and charge density differences of non-periodic bilayers BiOCl with HH-stack, AH-stack, AA-stack?*

Reply: Following the referee's suggestion, we have added plots showing the densities of states and charge density differences of the bilayers BiOCl with HH-stack, AH-stack, AA-stack as the new Fig. S19 (shown also as Fig. R6 below for the convenience of the referee). We note that the non-periodic incommensurate stacking of BiOCl bilayers in our MSL model is simulated by calculating the structures that represent the local behavior in different regions of the MSL under periodic boundary condition. We thank the referee for this suggestion as these plots help illustrate the

changes in the interlayer interaction with structure in different regions and support our arguments in a more clear way.

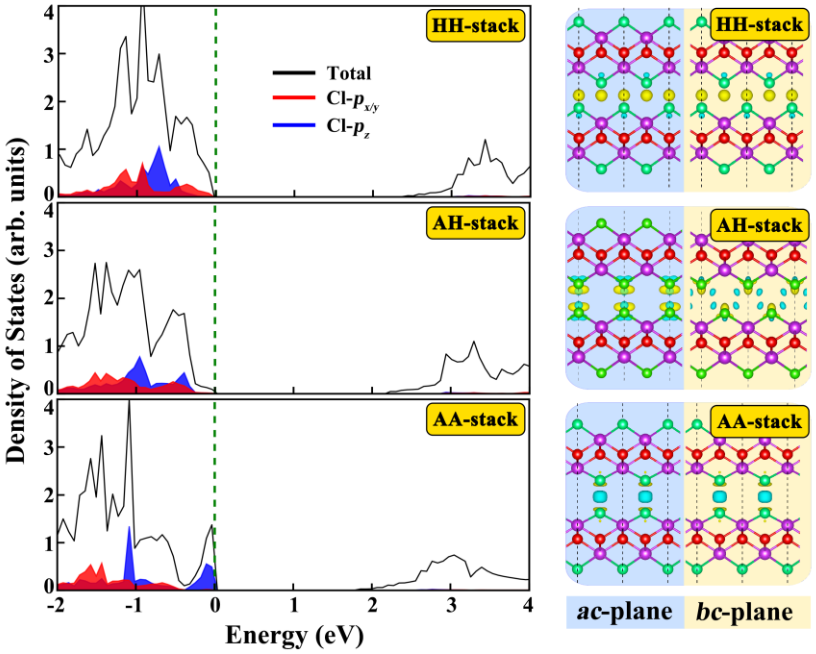

Figure R6: (Figure S19) The electronic densities of states (left panels) and charge density differences (right panels) of the BiOCl bilayers with HH-stack, AH-stack, AA-stack patterns under periodic boundary condition. For the charge density differences the isosurface value is 0.002 electrons/Å^3, and the charge accumulation and depletion are denoted in yellow and blue, respectively.

(8) Referee: *The bulk BiOCl has the ability to degrade RhB under visible light with dye sensitization of RhB. To avoid confusion, the authors should detect the visible-light photocatalytic activity of BiOCl MSLs in another way, for example, the degradation of colorless molecules.*

Reply: We thank the referee for this constructive suggestion. We have now measured the visible-light photocatalytic activity of BiOCl MSLs in the photodegradation process of colorless tetracycline (TC) antibiotic molecule. The results are shown in the new Fig. S26 (shown also as Fig. R7 below for the convenience of the referee). Compared with the bulk sample, the MSL clearly shows enhanced photocatalytic activity to degrade the colorless TC. Particularly, after 100 min visible light illumination, about 83.8% TC is degraded by the MSL, while only 12.1% TC is

degraded by the bulk. The corresponding discussions have also been added to p. 14 line 17 of the main text.

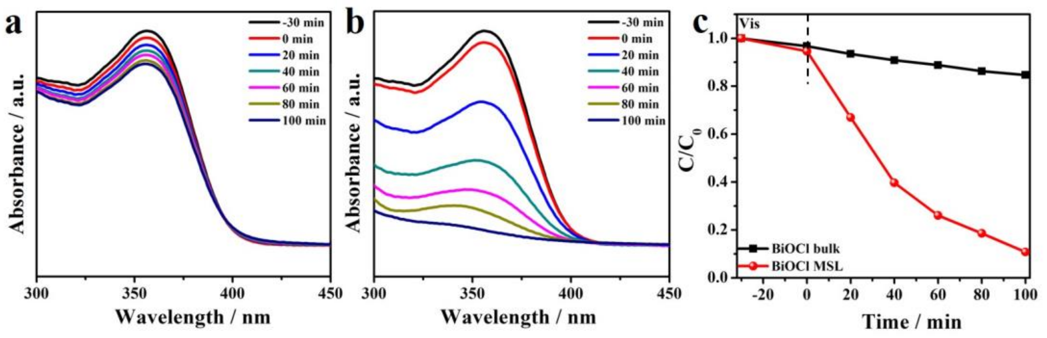

Figure R7: (Figure S26) Photocatalytic degradation of tetracycline with a) BiOCl bulk and b) BiOCl MSL nanosheets as catalysts. The tetracycline degradation is detected by the change in intensity of its characteristic peak of temporal UV-vis absorption spectrum. Measurements are taken under visible light irradiation. Negative time denotes dark condition before light irradiation. c) Photocatalytic degradation rate of tetracycline versus time with BiOCl bulk and BiOCl MSL nanosheets as catalysts.

(9) Referee: *In the experimental part, the BiOCl MSL was bottom-up chemical growth of spiral-type nanosheets with 1.6～3.0° twist angles among adjacent sheets, however, in the modeling section, the BiOCl spiral bilayer structure with the 1.7° twist angle was constructed, one was the adjacent sheet (each sheet consists of seven BiOCl atomic monolayers), and the other was the bilayer structure. This description made the modeling and the experiment seriously inconsistent. If this modeling is reasonable, the authors are recommended to change the description of bilayers to represent the bottom layer of the upper sheet and the top layer of the lower sheet for better understanding.*

Reply: This has been now clarified more explicitly in our revised text, Fig.1j, and newly added Fig.1k (see also our replies to the referee's comments #2 and #3). We appreciate the referee for offering us the opportunity to improve the clearness of our presentation in the manuscript.

Reviewer #2

(1) Referee: *The authors report on the synthesis of bismuth oxychloride spiral nanosheets with small interlayer twist angles using a facile chemical approach. The resultant Moiré superlattice structure lead to changes in their properties as compared to bulk HH-stacked structure such as reduced bandgap, enhanced carrier lifetime and photocatalytic activity. Overall this work has interesting results which broaden the understanding of screw-dislocation driven growth of 2D nanomaterials and probed into their unique properties. Based on novelty and the results gathered, the reviewer will consider publication after the authors consider these questions:*

Reply: We thank the referee for the above positive comments, and for the below constructive suggestions that help us improve the manuscript quality.

(2) Referee: *There is a lack of introduction to BiOCl and other 2D materials having screw-dislocation driven growth. What is the current research status of BiOCl nanosheets? (i.e., synthesis methods, properties and applications) This would make the manuscript much more comprehensive to the wider field.*

Reply: By following the referee's good suggestion, we have now added the following texts on the introduction to BiOCl in p. 4 line 5 of the revised manuscript:

"As a stable layered wide-gap semiconductor, bismuth oxychloride (BiOCl) has received great attention owing to its optical and electrical properties feasible for photocatalysis applications, such as water splitting[1], dye degradation[2], N_2 reduction[3], etc. With the aim of enhancing photocatalytic activity, various nanocrystals including 2D nanosheets have been synthesized by hydrolysis process, hydrothermal/solvothermal and ionic-assisted methods[4]."

For other 2D materials having screw-dislocation driven growth, the following texts are added in p. 8 line 1:

"The 2D spiral nanosheets grown with the screw-dislocation driven mechanism have been reported in Au[5], transition metal dichalcogenides[6], bimetallic hydroxides[7], layered double hydroxides[8], metal diboride[9], metavanadate[10], etc."

Reference:

1. Cheng, H., Huang, B. & Dai, Y. Engineering BiOX (X = Cl, Br, I) nanostructures for highly efficient photocatalytic applications. Nanoscale 6, 2009-2026 (2014).
2. Guan, M., Xiao, C., Zhang, J., Fan, S., An, R., Cheng, Q., et al. Vacancy Associates Promoting Solar-Driven Photocatalytic Activity of Ultrathin Bismuth Oxychloride Nanosheets. Journal of the American Chemical Society 135, 10411-10417 (2013).
3. Li, J., Li, H., Zhan, G. & Zhang, L. Solar Water Splitting and Nitrogen Fixation with Layered Bismuth Oxyhalides. Accounts of Chemical Research 50, 112-121 (2017).
4. Di, J., Xia, J., Li, H., Guo, S. & Dai, S. Bismuth oxyhalide layered materials for energy and environmental applications. Nano Energy 41, 172-192 (2017).
5. Morin, S. A., Forticaux, A., Bierman, M. J. & Jin, S. Screw Dislocation-Driven Growth of Two-Dimensional Nanoplates. Nano Letters 11, 4449-4455 (2011).
6. Fan, X., Jiang, Y., Zhuang, X., Liu, H., Xu, T., Zheng, W., et al. Broken Symmetry Induced Strong Nonlinear Optical Effects in Spiral WS_2 Nanosheets. ACS Nano 11, 4892-4898 (2017).
7. Ni, B. & Wang, X. Edge overgrowth of spiral bimetallic hydroxides ultrathin-nanosheets for water oxidation. Chemical Science 6, 3572-3576 (2015).
8. Forticaux, A., Dang, L., Liang, H. & Jin, S. Controlled Synthesis of Layered Double Hydroxide Nanoplates Driven by Screw Dislocations. Nano Letters 15, 3403-3409 (2015).
9. Liu, D., Chu, Y., Ye, B. & Zhou, X. Spontaneous growth of hexagonal ZrB_2 nanoplates driven by a screw dislocation mechanism. CrystEngComm 20, 7637-7641 (2018).
10. Sun, Y., Wang, J., Chen, M., Li, C., Hu, Z. & Chou, S.-L. Screw dislocation-driven t-$Ba_2V_2O_7$ helical meso/nanosquares: microwave irradiation assisted-SDBS fabrication and their unique magnetic properties. Journal of Materials Chemistry C 5, 6336-6342 (2017).

(3) Referee: *(1) Are all the synthesized BiOCl spiral nanosheets formed with a small degree of interlayer twist angle? (2) Why is it only unique to BiOCl and not observed for pervious 2D materials or nanoplates with spiral structures (or this should be present for other 2D materials as well)?*

Reply: Yes, all our synthesized BiOCl spiral nanosheets formed with small degree of interlayer twist angles, ranging from 1.6° to 3.0°. To clearly illustrate this point, we have now added the low-magnification TEM image showing more our synthesized BiOCl spiral nanosheets as in the new Fig. S4 (shown also as Fig. R8 below for the convenience of the referee). As can be seen all the nanosheets show visible Moiré patterns, which are caused by the small degree of interlayer twist angles between two nanosheets.

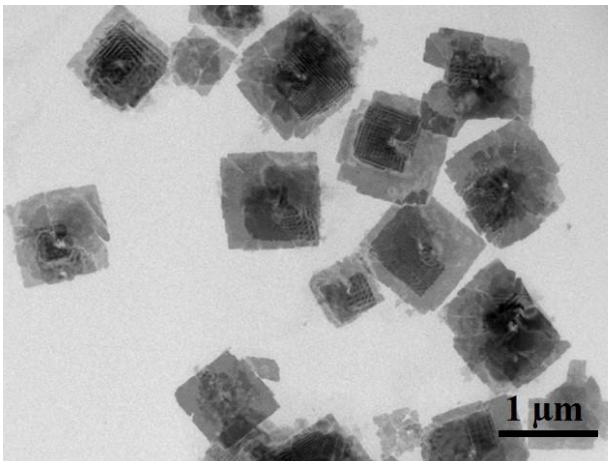

Figure R8: (Figure S4) Low-magnification TEM image of BiOCl MSL nanosheets.

As noted by the referee, the formation of interlayer twist angle between two nanosheets is essential for emergence of Moiré pattern, but not a sufficient condition. Among the synthesized 2D spiral nanosheets with screw-dislocation driven growth, the interlayer twist angle between two nanosheets was indeed reported, for instance in WS_2.[Fan et al., ACS Nano 11, 4892-4898 (2017)] But to our best knowledge, no observed Moiré patterns was reported yet. The origin may be ascribed to the unique characteristics of our synthesized BiOCl nanosheets, *i.e.*, high-quality, atomically thin, and large-area flat features and also suitable distance between the upper and lower sheets. These characteristics are favorable for the formation of Moiré pattern. We now add the following discussion in p. 8 line 1 of the revised manuscript:

"The 2D spiral nanosheets grown with the screw-dislocation driven mechanism have been reported in Au, transition metal dichalcogenides, bimetallic hydroxides, layered double hydroxides, metal diboride, metavanadate, etc. But we for the first time observe Moiré patterns in the current BiOCl spiral nanosheets. The origin may be ascribed to the fact that our synthesized BiOCl nanosheets have high-quality, atomically thin, and large-area flat features and also suitable distance between the upper and lower sheets, favorable for the formation of MSL."

(4) Referee: *(3) Since the formation of interlayer twist is important here, its mechanism should be explained. (4) Could this be associated to the strain generated by the screw-dislocation similar to "Eshelby twist" for 1D nanowires?*

Reply: In response to the referee's comment, we have now supplemented a schematic plot showing the growth mechanism of the spiral BiOCl nanosheets with interlayer twist angle as the new Fig. S10 (shown also as Fig. R9 below for the convenience of the referee). As in usual solvothermal reactions, isolated irregular nanosheets are formed at the preliminary stage (step I). During the assembly of isolated nanosheets, the line of screw dislocation forms. Under low supersaturation condition, which is the key to grow spirals [see Morin et al., Science 328, 476–480 (2010) and Meng et al., Accounts of Chemical Research 46, 1616-1626 (2013)], the screw dislocation line creates step edge upon intersection with a crystal surface (step II). Then, more reaction atoms are added on the step edge, and a new surface layer is created without need to overcome the energy barrier (step III). Finally, the spiral Hanoi tower-like structures are formed along the screw-dislocation axis (step IV and V). This growth mechanism is described in the known the Burton-Cabrera-Frank model[11,12]. In the process, the low supersaturation is essential for the screw dislocation to promote spiral crystal growth. Under the high supersaturation condition, the layer-by-layer dendritic growth mode is activated, which usually yields crystals with rough surfaces and fractal patterns. Accordingly, we have added the above description on the growth mechanism of spiral nanosheets into p. 7 line 19 of the main text and the revised Supplementary Information.

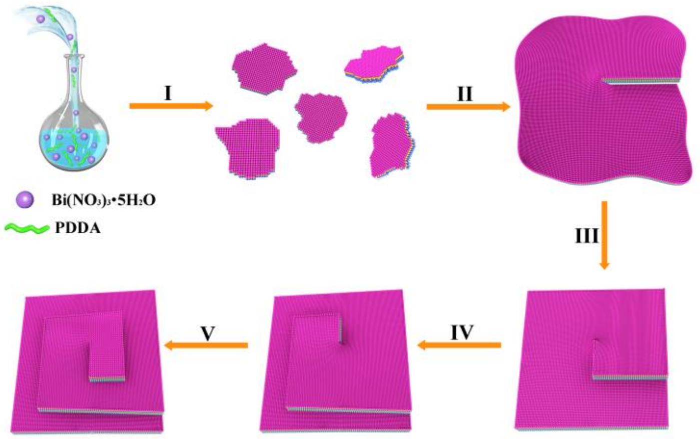

Figure R9: (Figure S10) Schematic plot showing the growth process of the spiral BiOCl MSL.

This screw-dislocation driven growth of 2D spiral nanosheets under low supersaturation condition shares the same mechanism with the “Eshelby twist” growth for 1D nanowires, which we appreciate the referee for bringing to our attention. The difference lies in the different step growth rates between different growth fronts, which result in different dimensional morphologies [see Meng et al., Accounts of Chemical Research 46, 1616-1626 (2013)].

To make this point clear, we have added the following discussion to p. 7 line 21 of the revised manuscript:

“The growth of spiral nanosheets with interlayer twist angle is attributed to the screw-dislocation driven self-perpetuating growth of spiral nanomaterials under the low supersaturation condition, as described by the Burton-Cabrera-Frank model[11,12]. This shares the same mechanism with the “Eshelby twist” growth for 1D nanowires[13].”

Reference:

11. Morin et al., Science 328, 476–480 (2010).
12. Burton, W. K., Cabrera, N. & Frank, F. C. Role of Dislocations in Crystal Growth. Nature 163, 398-399 (1949).
13. Meng, F., Morin, S. A., Forticaux, A. & Jin, S. Screw Dislocation Driven Growth of Nanomaterials. Accounts of Chemical Research 46, 1616-1626 (2013).

(5) Referee: *In Figure 2b, there are some peaks missing in their XRD spectra for 40 min and above. What does the missing peaks indicate?*

Reply: Further analysis on the XRD spectra motivated by the referee’s comment indicates that the missing peaks are caused by the emergence of Moiré pattern. As in the snap-shot TEM images (Fig. 2a), 40 min is the reaction time at which the Moiré pattern become observable. The formation of Moiré pattern in the incommensurate stacking of two nanosheets with twist angle breaks in-plane crystal periodicity. This loss of in-plane ordering is responsible for the corresponding peaks missing in the XRD spectra. Since in the vertically stacked nanosheets the periodicity along the out-of-plane direction is not changed whether Moiré patterns form or not, the corresponding peaks [such as (001), (002), (003)] are maintained. We now explicitly

discuss this in p. 8 line 9 of the revised manuscript. We thank the reviewer for the comment which helps us improve the discussion.

(6) Referee: *The term "Real-time" in the heading "Real-time characterization of growth…" is misleading. TEM was done after synthesis on transferred samples, not in "real-time.*

Reply: We have changed the heading to "Morphology characterization of growth of Moiré superlattices".

(7) Referee: *For multiple spiraling layers besides two layers, is there a constant interlayer twist between the successive layers? For example, does the periodicity of the Moiré pattern decreases with increasing layers? What effect does this have towards its emergent properties?*

Reply: We thank the referee for making this insightful comment. In response, we have added the following discussion in p. 15 line 17 of the revised manuscript.

"Finally, our synthesized MSLs are formed by the stacking of two spiraling nanosheets with one twist angle. It is possible to synthesize multiple spiraling nanosheets with the constant or changed twist angles between two adjacent sheets. The former will result in multiple MSLs stacked along the perpendicular direction with the same periodicity, whereas the latter will give rise to the MSLs with the changed periodicity. For such reassembly of the MSLs, the optical spectral properties will be contributed by individual MSLs, and there could be a gradient in properties such as bands edges, carrier concentration depending on the details."

Reviewer #3

(1) Referee: *In this work, 2D Moiré superlattices (MSLs) BiOCl was synthesized via solvothermal reaction, which was considered to follow a screw-dislocation driven mechanism. The fabrication of BiOCl MSLs leads to strong modulation of optoelectronic properties with 0.6 eV band gap reduction and substantial carrier lifetime increase, which may cause the enhanced visible-light photocatalytic activity. First-principles calculations further revealed that the locally enhanced inter-layer coupling effect associated with the Moiré potential modulation may play an important role in the modulation of optoelectronic properties. Only after addressing the following questions can this work be published.*

Reply: We thank the referee for the above positive comments on our work.

(2) Referee: *I am wondering if surfaces of BiOCl are clean enough, without capping agents residuals. If PDDA caps on the surfaces of BiOCl, how to remove it? Whether PDDA participate in catalytic process?*

Reply: The BiOCl nanosheet products were thoroughly washed with water three times to remove any residual reagent from them. In response to the referee's comment, we have now employed the XPS analysis technique to confirm the clean nature of nanosheet surfaces. The N 1s spectrum result is shown in the newly added Fig. S3 (shown also as Fig. R10 below for the convenience of the referee). There is no observable signal, which indicates the absence of any residual reagent such as PDDA on nanosheet surfaces. We have now added the statement on the clean nature of the BiOCl nanosheet surfaces in p. 5 line 18 of the revised manuscript.

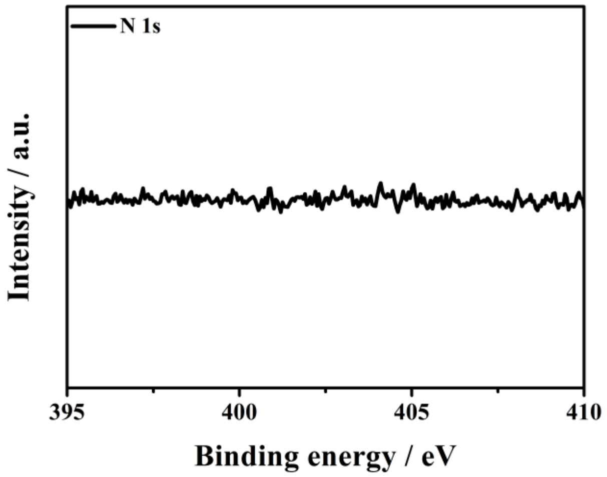

Figure R10: (Figure S3) N 1s XPS spectrum of BiOCl MSL nanosheets.

(3) Referee: *There are many methods to modify BiOCl, such as doping and heterostructures regulation. Compared to the traditional methods, what is the unique advantage of screw dislocation?*

Reply: As correctly pointed out by the referee, there are indeed many methods reported to modify BiOCl, such as doping with impurities, encapsulation involving metal nanoparticles, or heterostructures with other semiconductors, etc. While these strategies can modify optoelectronic properties of BiOCl, they all introduce extra external impurities/materials to BiOCl. Formation of the heterogeneous materials may bring benefits to photocatalytic activity, but also potentially degrade the intrinsic photocatalytic performance of BiOCl. The unique advantage of our work is that by exploiting the idea of formation of Moiré superlattice in 2D layered materials, we provide a clean modification of optoelectronic properties in pristine BiOCl without introducing any external impurities/materials. Equally importantly, to our best knowledge, this is the first time that the Moiré superlattice was synthesized by a facile, scalable chemical method and exploited to give emergent chemical functionality rather than only physical behaviors. In response to the referee's comment, we have added pertinent discussion on the above into p. 15 line 7 of the revised manuscript.

(4) Referee: *For the growth mechanism, more quantified information is need. For example, the authors claim the low supersaturation condition responsible for the screw dislocation, then how low, or what is the benchmark? Why low supersaturation condition is important?*

Reply: In response to the referee's comment, we have now supplemented a schematic plot showing the growth mechanism of the spiral BiOCl nanosheets as the new Fig. S10 (shown also as Fig. R11 below for the convenience of the referee). As in usual solvothermal reactions, isolated irregular nanosheets are formed at the preliminary stage (step I). During the assembly of isolated nanosheets, the line of screw dislocation forms. Under low supersaturation condition, which is the key to grow spirals [see Morin et al., Science 328, 476–480 (2010) and Meng et al., Accounts of Chemical Research 46, 1616-1626 (2013)], the screw dislocation line creates step edge upon intersection with a crystal surface (step II). Then, more reaction atoms are added on the step edge, and a new surface layer is created without need to overcome the energy barrier (step III). Finally, the spiral Hanoi tower-like structures are formed along the screw-dislocation axis (step IV and V). This growth mechanism is described in the known the Burton-Cabrera-Frank model[1,2]. In the process, the low supersaturation is essential for the screw dislocation to promote spiral crystal growth. Under the high supersaturation condition, the layer-by-layer dendritic growth mode is activated, which usually yields crystals with rough surfaces and fractal patterns. Accordingly, we have added the above description on the growth mechanism of spiral nanosheets into p. 7 line 19 of the main text and the revised Supplementary Information.

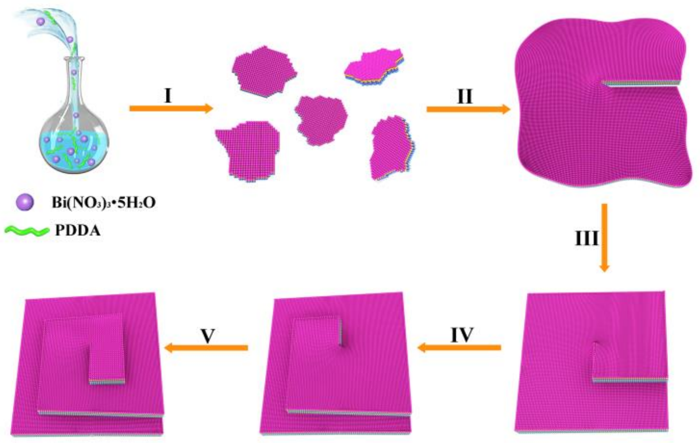

Figure R11: (Figure S10) Schematic plot showing the growth process of the spiral BiOCl MSL.

Regarding the referee's question on the benchmark of low supersaturation condition, we performed more synthesis trials by changing the concentration of $Bi(NO_3)_3$ and PDDA, as shown in the new Fig. S11 (shown also as Fig. R12 below for the convenience of the referee). We found that the spiral nanosheets of BiOCl cannot be obtained when the concentration of $Bi(NO_3)_3$ is higher than 0.4 mM and the concentration of PDDA higher than 2.25 wt%. One clearly sees in Fig. R11d that at the high concentration of 1.6 mM $Bi(NO_3)_3$ and 9 wt% PDDA, dendritic phase is formed. In response, we have added the above information on the benchmark of low supersaturation condition into p. 7 line 25 of the revised manuscript.

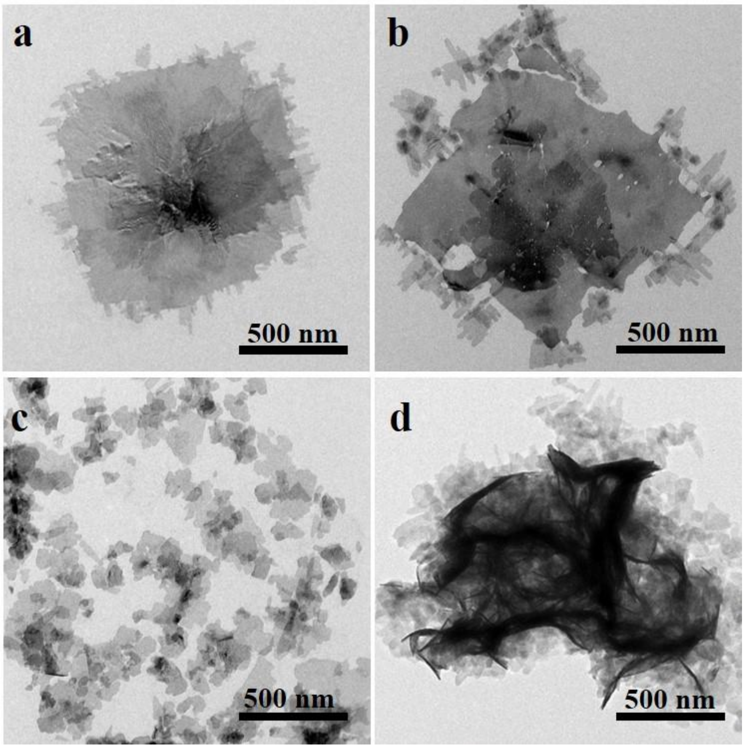

Figure R12: (Figure S11) a-d) Typical TEM images of the products obtained with the concentrations of a) $Bi(NO_3)_3$ (0.4 mM), PDDA (2.25 wt%) b) $Bi(NO_3)_3$ (0.8 mM), PDDA (4.5 wt%) c) $Bi(NO_3)_3$ (1.2 mM), PDDA (6.75 wt%) and d) $Bi(NO_3)_3$ (1.6 mM), PDDA (9 wt%).

Reference:

1. Morin et al., Science 328, 476–480 (2010).
2. Burton, W. K., Cabrera, N. & Frank, F. C. Role of Dislocations in Crystal Growth. Nature 163, 398-399 (1949).

(5) Referee: *TEM images in the manuscript just exhibited one single BiOCl nanosheet. TEM images that show more nanosheets are needed to further prove the monodispersity. Moreover, some cracks and irregular edges appeared in TEM images. What caused the existence of this kind of structure?*

Reply: Following the referee's good suggestion, we now add the low-magnification TEM image showing more our synthesized nanosheets to further prove the monodispersity as in the new Fig. S4 (shown also as Fig. R13 below for the convenience of the referee). From the low-magnification TEM image, we can see that all the nanosheets show pronounced Moiré patterns, which are caused by the small

degree of interlayer twist angles between two nanosheets. Regarding the cracks and irregular edges appeared in TEM images, we believe that they may be associated with the damage caused by the external forces during ultrasonic treatment and stirring in the process of sample preparation, or the result of relaxation of residual strains possibly existing in the nanosheets. We have now added this note into the caption of Fig. 1d in the revised manuscript.

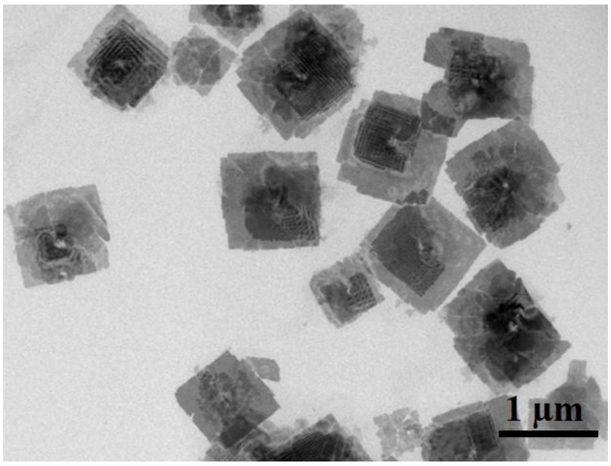

Figure R13: (Figure S4) Low-magnification TEM image of BiOCl MSL.

(6) Referee: *Cs-corrected HADDF STEM was widely used to characterize nanoscale structure down to atomic level. In this work, Cs-corrected HADDF-STEM should be used to characterize the differences among HH-stack, AH-stack, and AA-stack. What was the atomic arrangement in the uniaxial-oriented of BiOCl MSLs? Would strain exist in the uniaxial-oriented of BiOCl MSLs?*

Reply: We thank the referee for making this constructive suggestion. In response, we have now performed the aberration-corrected HADDF-STEM analysis on single spiral BiOCl nanosheet. The results are shown in the new Fig. S17 (shown also as Fig. R14 below for the convenience of the referee), in which we also show the structures from our theoretical simulation (Fig. R14d,g,h) for direct comparison. We can see that the Moiré patterns involving three distinct regions of HH-stack, AH-stack, AA-stack structures are observed in Fig. R14c. The results are in agreement with the structures from our theoretical simulation (Fig. R14d). From the high-resolution enlarged

HAADF-STEM image in Fig. R14e-f, we can see in detail the explicit atomic arrangements of the largest Bi atoms, which also agrees with the atomic structures in HH-stack and AH-stack region from the simulation results (Fig. R14g-h). We have now summarized this newly added results briefly in the main text (on p. 10 line 3).

Regarding the referee's question on the possibly existing strain in the uniaxial-oriented of BiOCl MSLs, we think the center of screw-dislocation and the edge of spiral nanosheets provide channels to mechanically relax residual strains. Therefore, the BiOCl MSLs should be free of strain distribution.

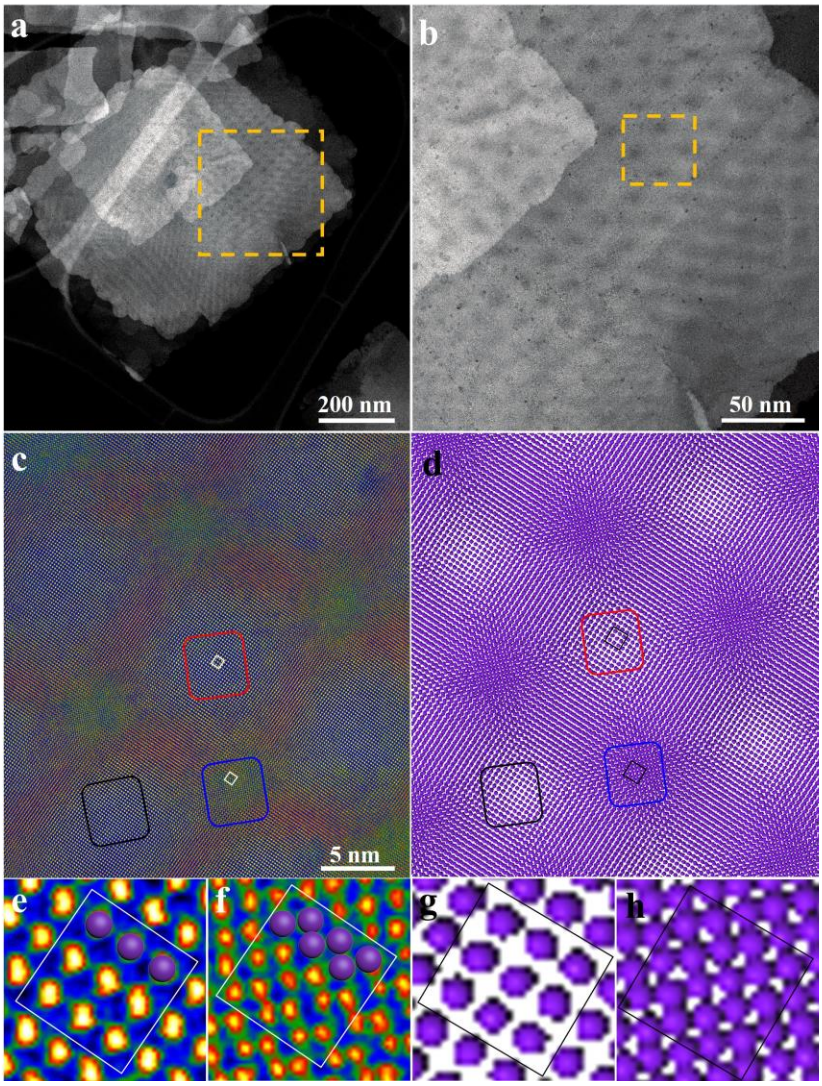

Figure R14: (Figure S17) a) Aberration-corrected HADDF-STEM image of BiOCl MSL. b) Amplified aberration-corrected HADDF-STEM image of selected region (in yellow) of (a) to clearly show profile of moiré pattern. c) The high-magnification contrast-false color HAADF-STEM image of

selected region (in yellow) of (b) to clearly observe three distinct regions (named HH-stack, AH-stack, AA-stack structures in Fig. 3a). d) Perpendicular view of our simulated BiOCl bilayer model showing the MSL structures with twist angle. For clarity only Bi atoms are shown. e, g) Amplified contrast-false color HAADF-STEM image and simulated BiOCl bilayer model image in the square area of red square of (c), (d), respectively. f, h) Amplified contrast-false color HAADF-STEM image and simulated BiOCl bilayer model image in the square area of blue square of (c), (d), respectively.

(7) Referee: *I am wondering if high-indexed facets could exist on step sites in the spiral nanosheets. If so, please index the specific facets.*

Reply: To address the referee's comment, we have also employed the aberration-corrected HADDF-STEM measurement focusing on the nanosheet edge regions. The results are shown in the new Fig. S18 (shown also as Fig. R15 below for the convenience of the referee). This allows us to clearly resolve atomic-scale structures on the step sites to detect the possible existence of high-indexed facets. As anticipated by the referee, high-indexed facets indeed exist near the nanosheet edge, which is indicated by the appearance of high density of low-coordinate (003) atomic steps, and few cases of (101) atomic steps. We have now mentioned this point in p. 10 line 7 of the revised manuscript.

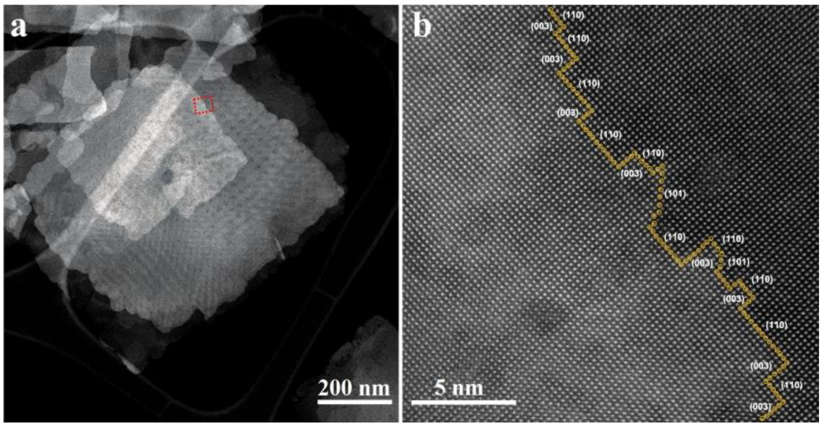

Figure R15: (Figure S18) a) Aberration-corrected HADDF-STEM image of BiOCl MSL. b) Amplified aberration-corrected HADDF-STEM image of selected region (in red) of (a) to clearly show the crystal faces on the step sites.

(8) Referee: *The authors mentioned bidirectional growth with AFM images. Are the growth speeds in two directions the same?*

Reply: Our BiOCl nanosheets are synthesized via the solvothermal reaction in solution. According to what we have observed, the growth speeds in two directions for the bidirectional growth of spiral nanosheets are same. Though we do not have real-time monitoring techniques for the synthesis procedure, the same-speed bidirectional growth of spiral nanosheets is indirectly supported by the steady-state STEM images of the final products (Fig.1c).

(9) Referee: *The manuscript discussed HH-stack and AA-HH-stack a lot, but did not pay too much attention on the HA-stack structure. Could the authors explain the unique properties of HA stack and how it worked in this system?*

Reply: As one of three predominated structures constituting the BiOCl MSL, the HA-stack structure can actually be seen as the intermediate structure between the AA-stack and HH-stack structures, as shown in Fig. 3c. In response to the referee's comment, we have now supplemented more calculated results on the HA-stack structure, *i.e.*, the dependence of band gap on the interlayer distance in Fig. 3d (shown also as Fig. R16 below for the convenience of the referee). We can see that the HA-stack structure demonstrates a modest band gap change with varying interlayer distance, falling between those of AA-stack and HH-stack structures. This is also the case for other properties. Within the whole BiOCl MSL, the HA-stack structure is expected to make its contribution to the total optical spectrum, but not determine the optical threshold (band gap). Meanwhile, the HA-stack structure will contribute to the emerging gradient in properties such as bands edges alignment, carrier concentration and separation depending on spatial distribution of the three stacking structures in the MSL. Pertinent discussion on the above has been added into p. 11 line 13 of the revised manuscript.

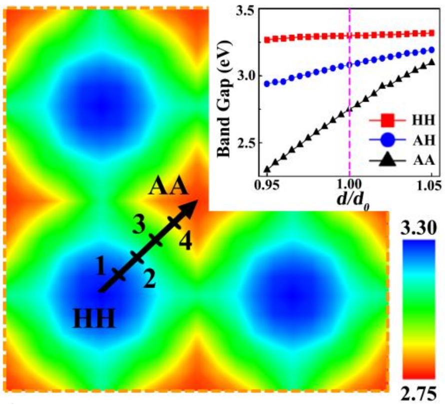

Figure R16: (Figure 3d) Variation of calculated band gaps throughout the MSL, *E*g(*r*), shown within the yellow squared region of (a). The inset shows the dependence of band gap on the interlayer distance *d* for the HH-stack, AH-stack, AA-stack structures. d_0 = 0.736 nm represents the experimentally determined distance between the two adjacent nanosheets.

(10) Referee: *Whether the L-edge of XANES and EXAFS for Bi in spiral nanosheets and bulk BiOCl are the same?*

Reply: To address the referee's question, we performed new characterization of the L_{III}-edge of XANES and EXAFS for Bi in BiOCl spiral nanosheet and bulk sample. The results are shown in the new Fig. S22 (shown also as Fig. R17 below for the convenience of the referee). From Fig. R17a, we see that the Bi L_{III}-edge XANES profiles in spiral nanosheet and bulk sample are nearly identical, consisting with the same chemical state of Bi in different phases. For the Bi L_{III}-edge EXAFS profiles (Fig. R17b), the intensities of Bi-O peak and Bi-Cl peak of spiral nanosheet are slightly lower than those of bulk sample. This may be attributed to the emergent local disorder of atomic coordination environment in the spiral nanosheet associated with the existing screw dislocation and formation of Moiré pattern. Pertinent discussion on the above has been added into p. 12 line 25 and p. 13 line 1 of the revised manuscript.

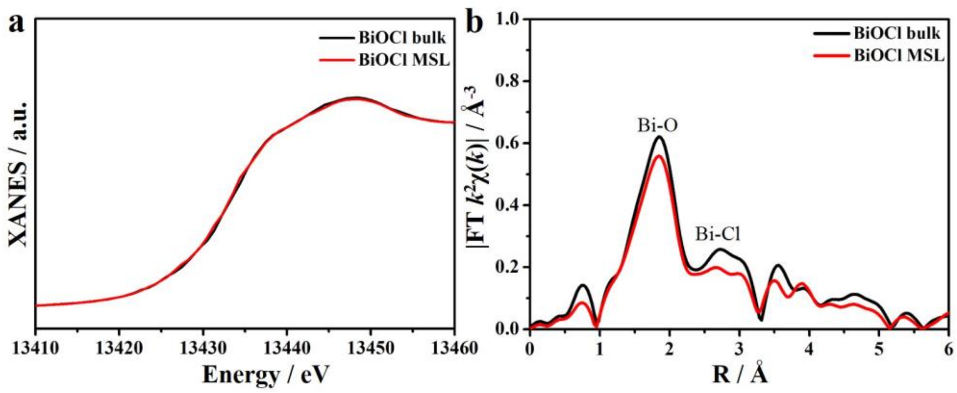

Figure R17: (Figure S22) a) The normalized XANES spectra of different samples at the Bi L_{III}-edge. b) The Bi L_{III} -edge EXAFS spectra of different samples.

(11) Referee: *What are the reaction sites on BiOCl MSLs during catalytic degradation, the oriented axis, high-indexed facets, edge sites, or others?*

Reply: We thank the referee for making this insightful comment. Unfortunately, as the reviewer may know, the detection of explicit reaction sites during catalytic degradation is very challenging and beyond the scope of this study. There exist high-indexed facets near the nanosheet edge in our synthesized BiOCl spiral nanosheets. There are also screw dislocation axis and edge sites as mentioned by the referee. These are potential reaction sites during catalytic degradation. We now explicitly state these possibilities, with the comment that future work to distinguish them will be of interest (in p. 14 line 23 of the main text).

Reviewers' comments:

Reviewer #1 (Remarks to the Author):

The authors have done a substantial revision and the quality of the paper was improved. However, there were still critical issues remaining.
1.Previously, the authors mistook the distance of BiOCl c axis (0.736 nm) for the distance between the two adjacent MSL nanosheets. In the last revision, the authors revised this point in Figure 1 but did not correct it in Figure 3d, i.e., there was no experimental evidence to demonstrate the distance of AA-stack. Since the lack of such experimental result would make the calculation results in Figure 3 meaningless in terms of bandgap determination, the authors are strongly recommended to provide a side-view HRTEM image of BiOCl MSL to show the distance between the two adjacent MSL nanosheets.
2.It is recommended to change the description of bilayers to represent the bottom layer of the upper sheet and the top layer of the lower sheet in the modeling section, rather than "the inner BiOCl layers of the nanosheets do not contribute to such spatial modulation". This argument is questionable, even wrong.
3.Along the orientation axis, a large number of defect structures (low-coordinated sites) appear, which can also promote photocatalytic activity of the materials. It do not exclusively stem from the changes in optical properties mentioned above. From the TEM images, we can roughly discern that the area of the central screw - dislocation region of the BiOCl MSL (30 min) is substantially equal to that of the BiOCl MSL (120 min). The difference between the two samples is mainly focused on coverage percent of the MSL. If the two samples are put together for comparison, the impact caused by the introduction of defect structures should be ruled out to a great extent. So the photocatalytic activity data of these two samples should be provided. As for the edge step sites mentioned by other reviewers, their contribution to the activity is not so great.
4.The UV/vis absorption spectra of spiral BiOCl nanosheets possessed obvious trailing phenomenon caused by defects, however, the EPR spectrum of BiOCl did not reveal the existence of defects. The authors need comment on this point.

Reviewer #2 (Remarks to the Author):

The authors had clarified all our questions and had made the appropriate revisions. Therefore, I would recommend publication of this paper.

Reviewer #3 (Remarks to the Author):

The revision has been made accordingly to my comments and improved significantly. I recommend acceptance of this paper in this form.

Point-by-point Responses to Reviewers' Comments

Note: *The Reviewers' Comments are with italic*, our Responses are in blue, the new changes made in the manuscript are in red.

<u>Reviewer #1</u>

(1) Referee: *The authors have done a substantial revision and the quality of the paper was improved. However, there were still critical issues remaining.*

Reply: We thank the referee for making further constructive and specific comments to help us improve the quality of our work.

(2) Referee: *Previously, the authors mistook the distance of BiOCl c axis (0.736 nm) for the distance between the two adjacent MSL nanosheets. In the last revision, the authors revised this point in Figure 1 but did not correct it in Figure 3d, i.e., there was no experimental evidence to demonstrate the distance of AA-stack. Since the lack of such experimental result would make the calculation results in Figure 3 meaningless in terms of bandgap determination, the authors are strongly recommended to provide a side-view HRTEM image of BiOCl MSL to show the distance between the two adjacent MSL nanosheets.*

Reply: We thank the referee for this good suggestion. We note that the actual distance between the two adjacent nanosheets was determined from the joint analysis of the AFM step height (Supplementary Fig. 1) and TEM data (Fig. 1i). It is equal to the length of *c* axis for BiOCl lattice (0.736 nm) as noticed by the referee. Following the referee's suggestion, we have now performed the side-view HRTEM measurement of BiOCl MSL. The cross section of the MSL was obtained by using the focused ion beam, in which procedure the measured micro area is protected by deposited platinum before being etched by gallium ions. The results are shown in the newly added Supplementary Fig. 9 (shown also as Fig. R1 below for the convenience of the referee). We observe straightly and clearly that the distance between the two adjacent nanosheets is equal to the length of c axis for BiOCl lattice.

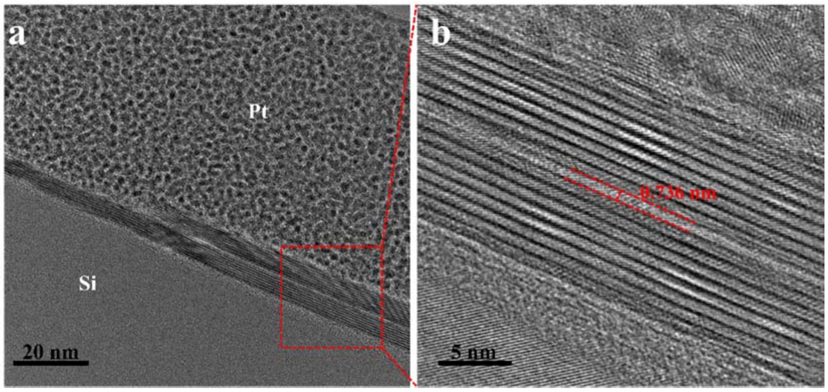

Fig. R1 (Supplementary Fig. 9): The side-view HRTEM image of BiOCl MSL nanosheet. The cross section of the MSL was obtained by using the focused ion beam, in which procedure the measured micro area is protected by deposited platinum before being etched by gallium ions.

Accordingly, in p. 7 lines 174-176 of the revised manuscript we have now added the following discussion:

"The side-view HRTEM image (where the cross section of the MSL was obtained by using the focused ion beam, Supplementary Fig. 9) further indicates that the distance between the two adjacent nanosheets is equal to the length of c axis for BiOCl lattice."

(3) Referee: *It is recommended to change the description of bilayers to represent the bottom layer of the upper sheet and the top layer of the lower sheet in the modeling section, rather than "the inner BiOCl layers of the nanosheets do not contribute to such spatial modulation". This argument is questionable, even wrong.*

Reply: As suggested by the referee, in p. 7 lines 182-183 we have changed the sentence of "The inner BiOCl layers of the nanosheets do not contribute to such spatial modulation." to "We thus adopt the BiOCl bilayers to represent the bottom layer of the upper sheet and the top layer of the lower sheet."

(4) Referee: *Along the orientation axis, a large number of defect structures (low-coordinated sites) appear, which can also promote photocatalytic activity of the materials. It do not exclusively stem from the changes in optical properties mentioned*

above. From the TEM images, we can roughly discern that the area of the central screw - dislocation region of the BiOCl MSL (30 min) is substantially equal to that of the BiOCl MSL (120 min). The difference between the two samples is mainly focused on coverage percent of the MSL. If the two samples are put together for comparison, the impact caused by the introduction of defect structures should be ruled out to a great extent. So the photocatalytic activity data of these two samples should be provided. As for the edge step sites mentioned by other reviewers, their contribution to the activity is not so great.

Reply: We thank the referee for this insightful comment. In response, we have now provided direct comparison between the visible-light photocatalytic activity data of BiOCl MSL (30 min) and BiOCl MSL (120 min), as shown in the new Supplementary Fig. 26 (shown also as Fig. R2 below).

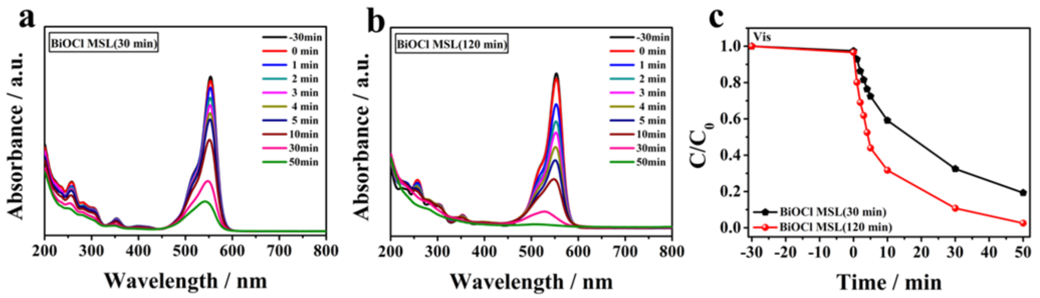

Fig. R2 (Supplementary Fig. 26): (a,b) Photocatalytic degradation of Rhodamine B with BiOCl MSL (30 min) and BiOCl MSL (120 min) nanosheets as catalysts. The Rhodamine B degradation is detected by the change in intensity of its characteristic peak of temporal UV-vis absorption spectrum. Measurements are taken under visible light irradiation. Negative time denotes dark condition before light irradiation. c) Comparison of photocatalytic degradation rate of Rhodamine B versus time for the two samples.

Accordingly, in p. 15 lines 392-401 of the revised manuscript we have now added the following discussion:

"It should be pointed out that the defect structures (*i.e.*, low-coordinated sites) around the screw-dislocation axis may also contribute to the promotion of photocatalytic activity of BiOCl MSLs. To exclude this effect we compare directly the visible-light photocatalytic activity data of the BiOCl MSLs (30 min) and BiOCl MSLs (120 min) (Supplementary Fig. 26), which have almost the same area of the

central screw-dislocation region, but different coverage percent of the MSL. The results show substantially enhanced photocatalytic activity in the BiOCl MSLs (120 min). This further evidences that the enhancement of photocatalytic activity is attributed to the emergence of MSL inducing strong modulation of optoelectronic properties as discussed above."

(5) Referee: *The UV/vis absorption spectra of spiral BiOCl nanosheets possessed obvious trailing phenomenon caused by defects, however, the EPR spectrum of BiOCl did not reveal the existence of defects. The authors need comment on this point.*

Reply: We have now added the following discussion in p. 9 lines 231-235 of the revised manuscript:

"We note that in spite of no EPR signal revealing the existence of defects, there appears an observable trailing behavior in the visible region of UV/vis absorption spectra (Supplementary Fig. 13). This may ascribe to the emergent defects caused by the sample surface adsorbed hydroxyl and the laser irradiation during UV/vis spectra measurement.[S. Wu, et al., Chem. Mater, 2018, 30, 5128−5136]"

Reviewer #2

(1) Referee: *The authors had clarified all our questions and had made the appropriate revisions. Therefore, I would recommend publication of this paper.*

Reply: We appreciate the referee for the valuable efforts in reviewing our manuscript.

Reviewer #3

(1) Referee: *The revision has been made accordingly to my comments and improved significantly. I recommend acceptance of this paper in this form.*

Reply: We thank the referee for the constructive comments that help us greatly improve the manuscript quality.

REVIEWERS' COMMENTS:

Reviewer #1 (Remarks to the Author):

The novelty of this work is the Moiré superlattice of BiOCl for band gap reduction, increase in carrier lifetime, and thus the enhanced photocatalytic activity. The first report of BiOCl Moiré superlattice is of great interest, however, the assignment of band gap reduction merely to the Moiré superlattice is risky owing to the presence of defects, as evidenced by Bi LIII-edge EXAFS spectra and DRS spectra of BiOCl MS. The authors have commented on the influence of defect to DRS spectra in the last revision, I think the manuscript could be accepted for publication after the authors comment on the possible influence of defect to band gap reduction, which would help the readers to get an unbiased knowledge of bandgap modulation for BiOCl.

Point-by-point Responses to Reviewers' Comments

Note: *The Reviewers' Comments are with italic*, our Responses are in blue, the new changes made in the manuscript are in red.

<u>Reviewer #1</u>

(1) Referee: *The novelty of this work is the Moiré superlattice of BiOCl for band gap reduction, increase in carrier lifetime, and thus the enhanced photocatalytic activity. The first report of BiOCl Moiré superlattice is of great interest, however, the assignment of band gap reduction merely to the Moiré superlattice is risky owing to the presence of defects, as evidenced by Bi LIII-edge EXAFS spectra and DRS spectra of BiOCl MS. The authors have commented on the influence of defect to DRS spectra in the last revision, I think the manuscript could be accepted for publication after the authors comment on the possible influence of defect to band gap reduction, which would help the readers to get an unbiased knowledge of bandgap modulation for BiOCl.*

Reply: We thank again the referee for all his/her valuable efforts to help us improve the manuscript. We agree with the referee on the possible influence of defects (caused by the sample surface adsorbed hydroxyl and the laser irradiation during UV/vis spectra measurement) to band gap reduction. We have now added the comment of "*The emergence of such defects may lead to band gap reduction to some extent, in addition to the predominated contribution from the formation of MSL as discussed.*" in p. 9 lines 224-226 of the revised manuscript:
